# Supplementary material for: Combined inhibition of BADSer99 phosphorylation and PARP ablates models of recurrent ovarian carcinoma
Source: Commun Med (Lond). 2022 Jul 2;2:82. doi: 10.1038/s43856-022-00142-3 (PMC9250505; doi:10.1038/s43856-022-00142-3)
Supplement: Supplementary file 1 — Supplementary Information [file 43856_2022_142_MOESM1_ESM.pdf]

Supplementary Information for

Combined inhibition of BADSer99 phosphorylation and PARP ablates models of recurrent ovarian carcinoma

Xi Zhang, Liqiong Wang, Peng Huang, Shu Chen, Lan Ma, Hui Ding, Basappa Basappa, Tao Zhu, Peter E. Lobie, and Vijay Pandey

Peter E. Lobie, Room1210, Information Building, Tsinghua Shenzhen International Graduate School, Nanshan, Shenzhen, 518055 P.R. China, Email: pelobie@sz.tsinghua.edu.cn
Vijay Pandey, Room1209, Information Building, Tsinghua Shenzhen International Graduate School, Nanshan, Shenzhen, 518055 P.R. China, Email: vijay.pandey@sz.tsinghua.edu.cn

This PDF file includes:

Supplementary Figures 1 to 11
Supplementary Tables 1 to 9
Supplementary Methods
Supplementary References

Supplementary Figure 1.

A. Optimal cut-off value based on the ROC curve in IRS for pBADS99 and BAD in NCO and EOC

	Groups	Cases	Staining score median	p	AUROC	p	Cut-off IRS value	Sensitivity	Specificity	Youden index
**pBAD**	EOC	80	6	0.00	0.791	0.00	3.5	0.875	0.6	0.475
	NCO	20	2.5							
**BAD**	EOC	80	3	0.00	0.934	0.00	5	0.95	0.8	0.75
	NCO	20	8							

B. ROC curve

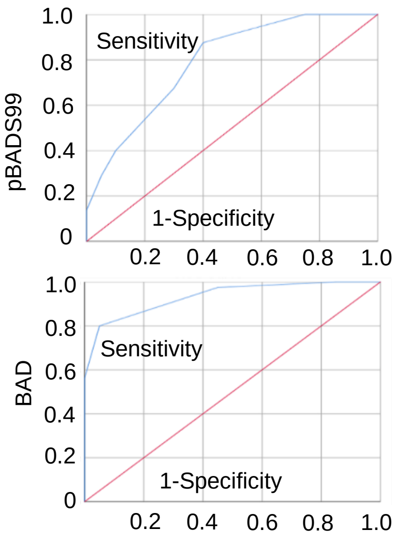

C. COX-regression model (pBADS99)

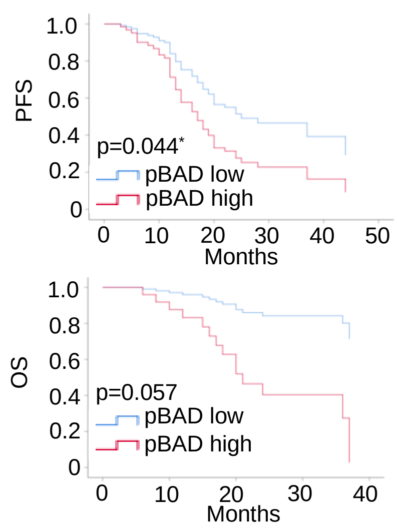

D. Kaplan-Meier curve (BAD)

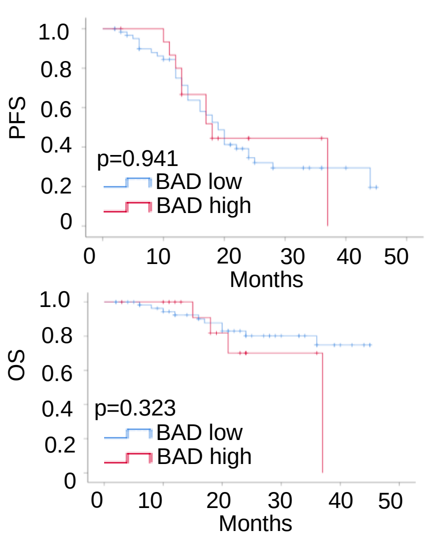

E. pBADS99 and EOC molecular status correlation

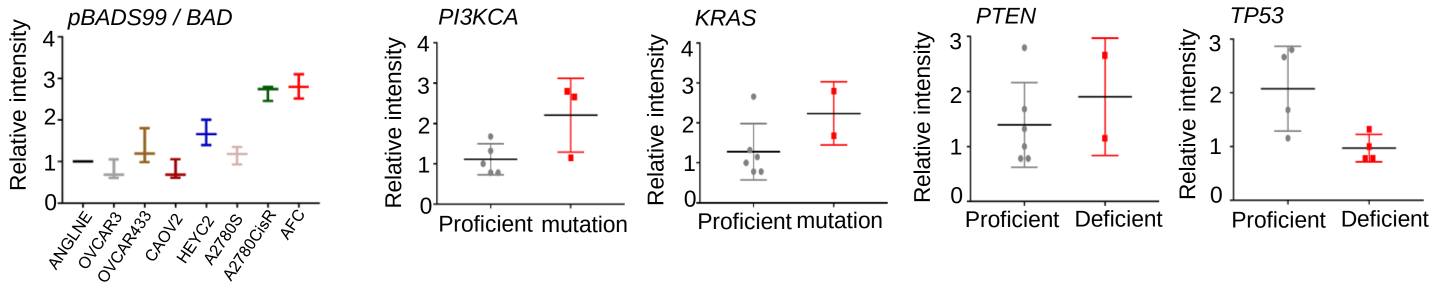

F. Correlation of pBADS99 to IC_{50} of Rucaparib or Talazoparib

Cell lines	IC_{50} ±SD (μM) Rucaparib (48h)	Talazoparib (72h)	Rucaparib (6 Days)	Talazoparib (6 Days)
Angline	1.00±0.05	1.0±0.05	0.25±0.08	0.14±0.003
OVCAR-3	1.87±0.02	3.60±0.85	1.48±0.36	2.16±0.48
OVCAR-433	6.73±1.0	2.40±0.78	6.03±2.04	0.90±0.37
CAOV-2 (OVCAR2)	2.50±1.87	1.40±0.58	1.14±0.33	0.24±0.05
HEYC2 (HEYA8)	4.60±0.78	3.90±1.43	1.20±0.39	0.79±0.22
A2780	4.30±1.5	2.10±1.20	1.63±0.42	0.096±0.003
A2780(cisR)	7.65±2.66	5.87±2.14	0.87±0.28	1.77±0.53
AFC	5.90±2.01	4.50±0.96	0.22±0.47	0.41±0.12

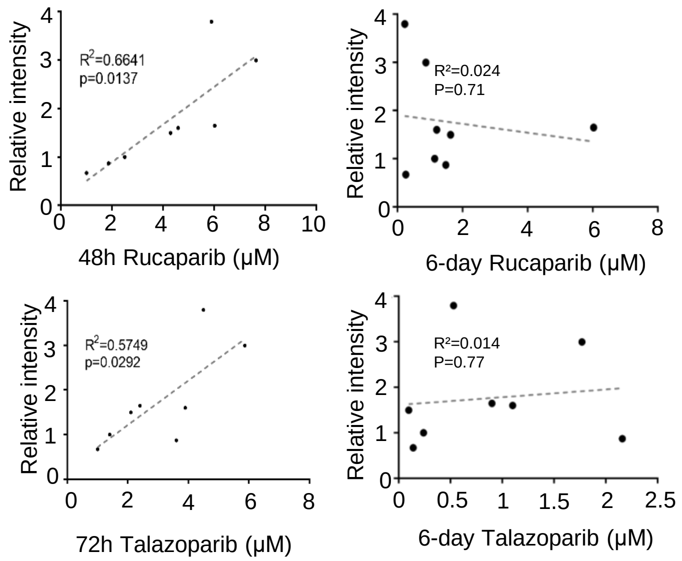

Supplementary Figure 1.

G*. Transient* transfection of *hBADS99A* in CAOV2 cells

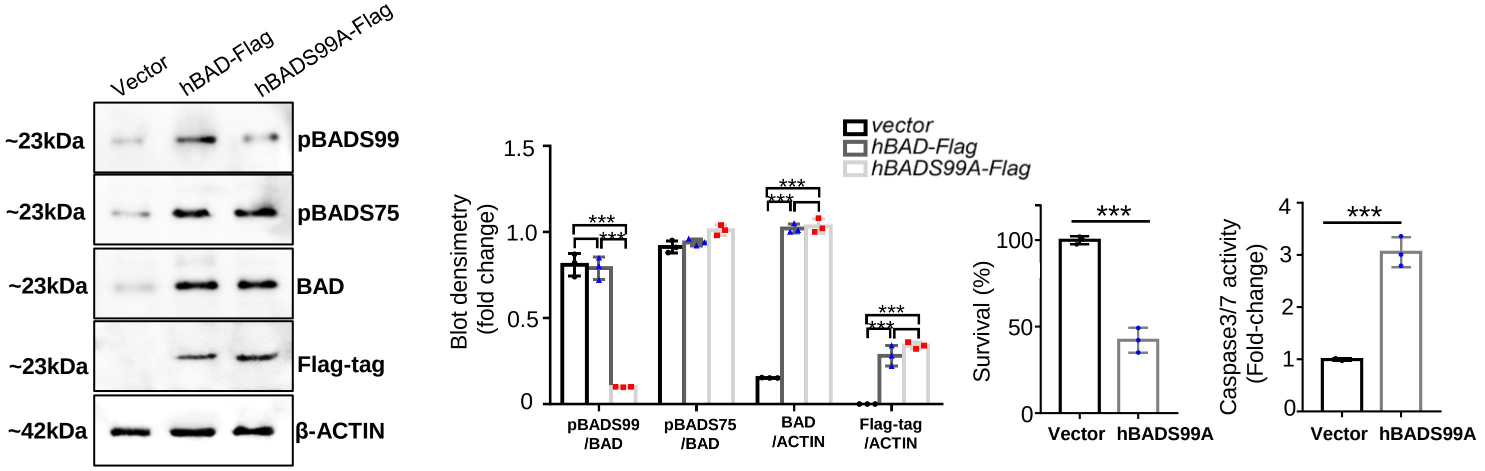

H. Confocal microscopy of tagged *hBAD-Flag* and *hBADS99A-Flag* construct in CAOV2

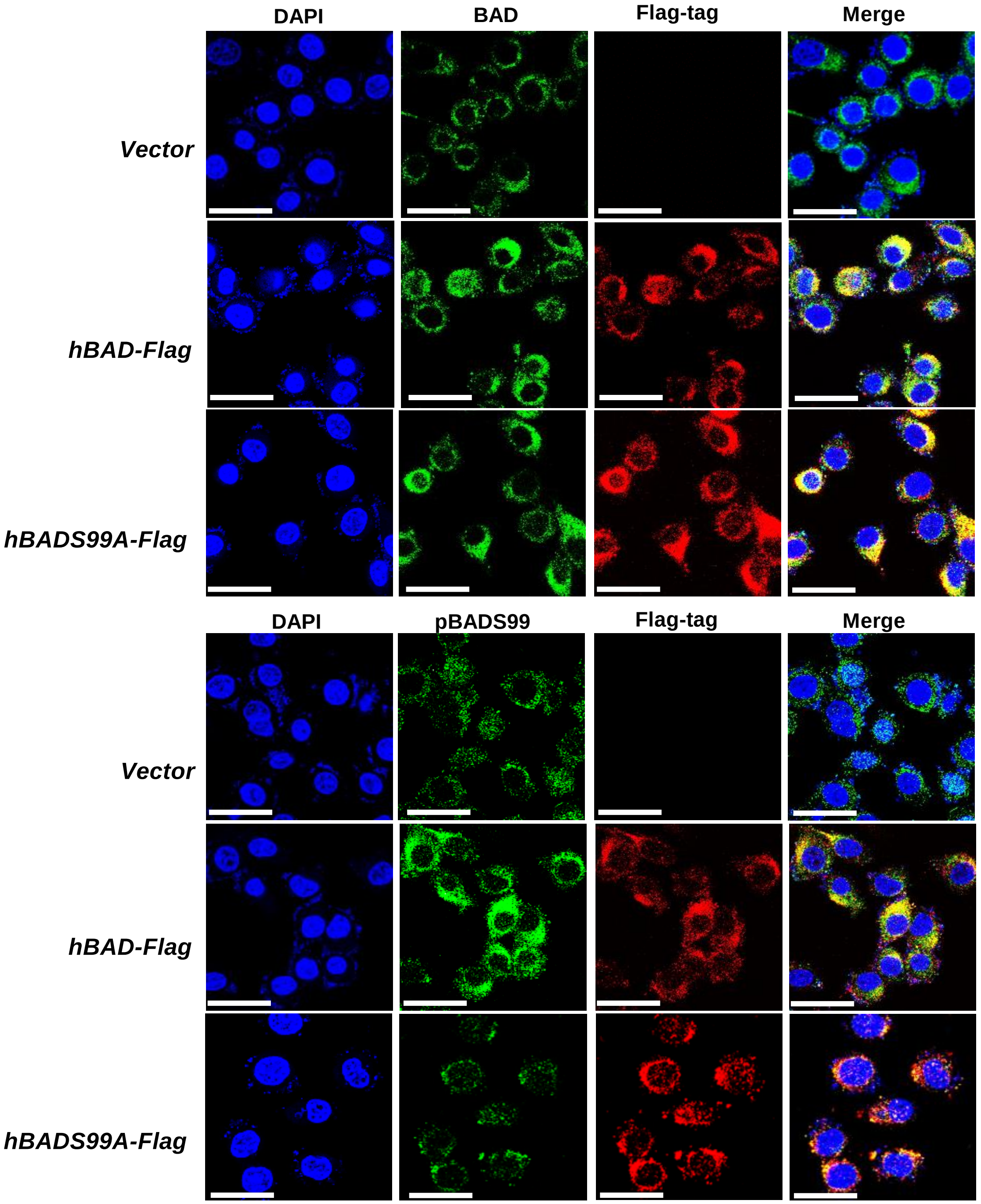

Supplementary Figure 1.

(A) Receiver operating characteristic (ROC) curve analyses were performed to calculate the optimal cut-off immunoreactive score (IRS) values and area under the receiver operating characteristics (AUROC) between low and high pBADS99 expression, based upon the maximal differences of sensitivity and specificity to discriminate epithelial ovarian cancer (EOC) tissue samples from non-cancer ovarian (NCO) tissue samples.

(B) Receiver operating characteristic (ROC) curve.

(C) Univariate analysis for survival outcome (overall survival (OS) and progression-free survival (PFS)) was performed using the Cox regression model to identify pBADS99 as an independent variable. Cox regression univariate analysis revealed worse PFS (p=0.044) rates for EOC patients with high pBADS99 expression compared with patients with low pBADS99. The immunoreactive score (IRS) 0 to 4 was categorized as low pBADS99 (n=29) and IRS 6 to 12 was categorized as high pBADS99 expression (n=51) in EOC patient cohort, as described in the methods section.

(D) Relapse and survival times were compared by Kaplan–Meier analysis and overall survival (OS) or progression-free survival (PFS) were tested for significance by using the log-rank test. The immunoreactive score (IRS) 0 to 4 categorized was categorized as low BAD (n=64) and IRS 6 to 12 as high BAD respectively (n=16) as described in the materials and methods section.

(E) Left: Relative intensity of pBADS99/BAD in 8 EOC cell lines. Right: No statistically significant association of pBADS99 with mutations of *PI3KCA, KRAS, PTEN, or TP53*) were observed in the panel of epithelial ovarian cancer (EOC) cells.

(F) Correlation between pBADS99 levels and Rucaparib or Talazoparib half-maximal inhibitory concentration (IC_{50}) values of EOC cell lines. Left: The IC_{50} of Rucaparib or Talazoparib in different time endpoints was calculated by total cell number counting (n=3). Right: levels of pBADS99 showed no correlation with IC_{50} of Rucaparib or Talazoparib sensitivity in EOC

cells: the IC_{50} of Rucaparib for 48h and the IC_{50} of Talazoparib for 72h exhibited a significant correlation with the levels of pBADS99 in EOC cells. Statistical changes were measured using *Pearson correlation* analysis between levels of pBADS99 and IC_{50} values of Rucaparib or Talazoparib in EOC cell lines.

(G) Forced expression of *Flag-hBAD* and *Flag-hBADS99A* in CAOV2 cells was confirmed by Western Blot analysis. Forced expression of *Flag-hBADS99A* in CAOV2 cells reduced cell viability and induced apoptosis. Cell viability and CASPASE 3/7 activity were evaluated using the ApoTox-Glo Triplex Assay Kit in CAOV2 cells 72 hours after transfection (n=3).

(H) Immunofluorescence staining was performed with a rabbit anti-BAD or pBADS99 and mouse monoclonal anti-Flag antibodies in Flag-tagged wild type *hBAD* and *hBADS99A* transfected CAOV2 cells. Scale bar, 50 μm.

Points are mean of triplicate experiments; bars, ±SD. **p <0.01, *** p <0.001.

Supplementary Figure 2.

Combination efficacy of NPB and PARP inhibitors in EOC cell lines

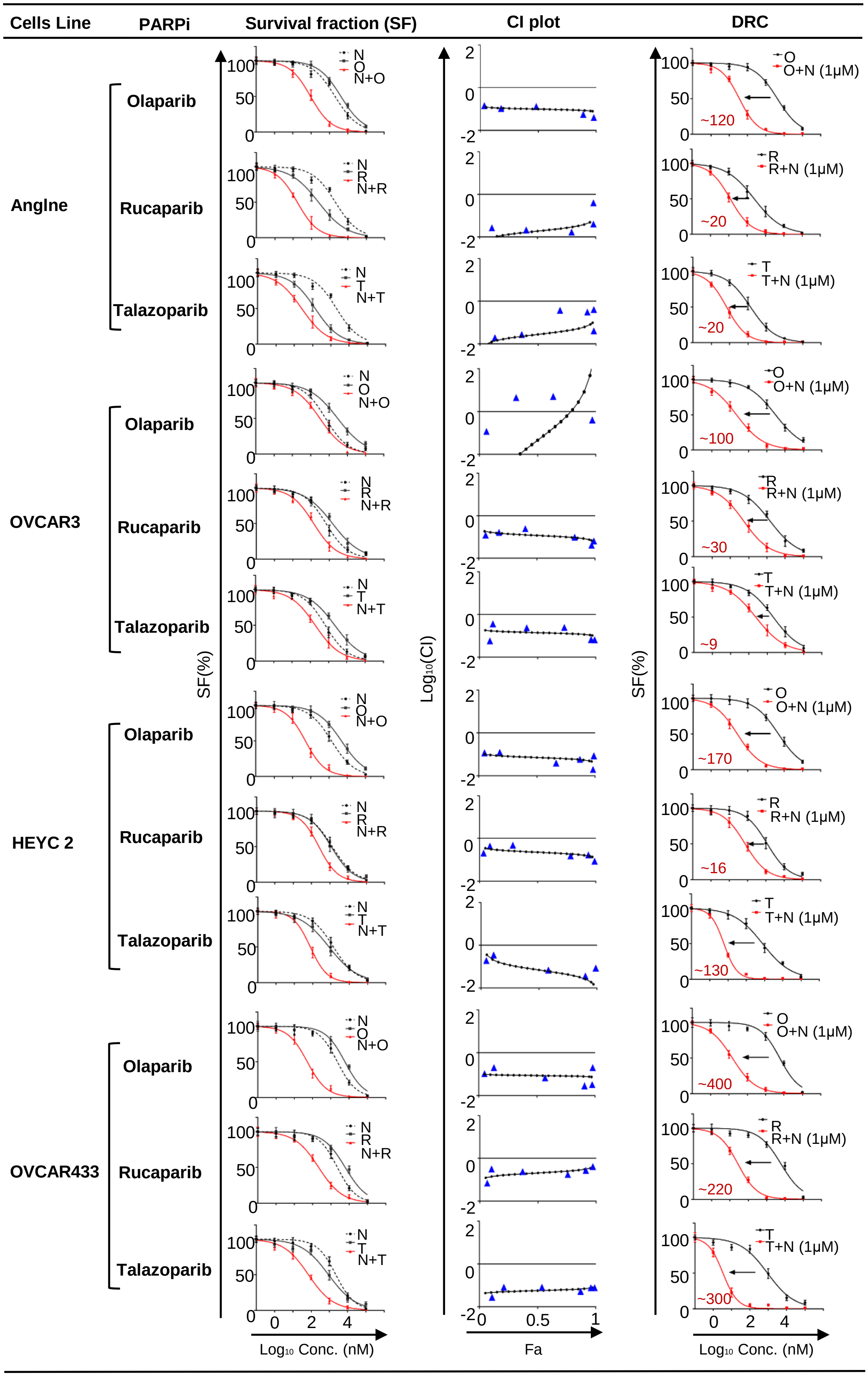

Supplementary Figure 2.

NPB synergizes with PARP inhibitors in EOC cells to decrease cell survival

Synergy (Chou-Talalay) analysis for NPB (N) in combination with three PARP inhibitors, namely Olaparib (O), Rucaparib (R), and Talazoparib (T), in a panel of EOC cells including Anglne (*BRCA-proficient* /Platinum sensitive), OVCAR3 (*BRCA-proficient* /Platinum sensitive), HEYC 2 (*BRCA-proficient* /Platinum sensitive) and OVCAR433 (*BRCA-deficient*/Platinum resistant). Cells were treated with the indicated concentration (Conc., $\log_{10}$ scale) of NPB and indicated PARP inhibitors for 6 days. The survival fraction (SF) was assessed using total cell number. The logarithmic combination index (CI) value corresponding to cell fraction affected (Fa) was determined using the CompuSyn software (http://www.combosyn.com) as described in materials and methods. CI value indicates: < 1 synergism; = 1 additive Synergy; > 1 antagonism (n=3). Dose-response curves (DRC) for a panel of cells treated with the indicated concentration of PARP inhibitors with or without 1uM NPB in a total cell number assay. Arrow indicates fold reduction in respective PARP inhibitor IC_{50} in the presence of NPB (n=3).

Points are mean of triplicate experiments; bars, ±SD.

Supplementary Figure 3.

A. CAOV2/CAOV2-*siRNA BAD*

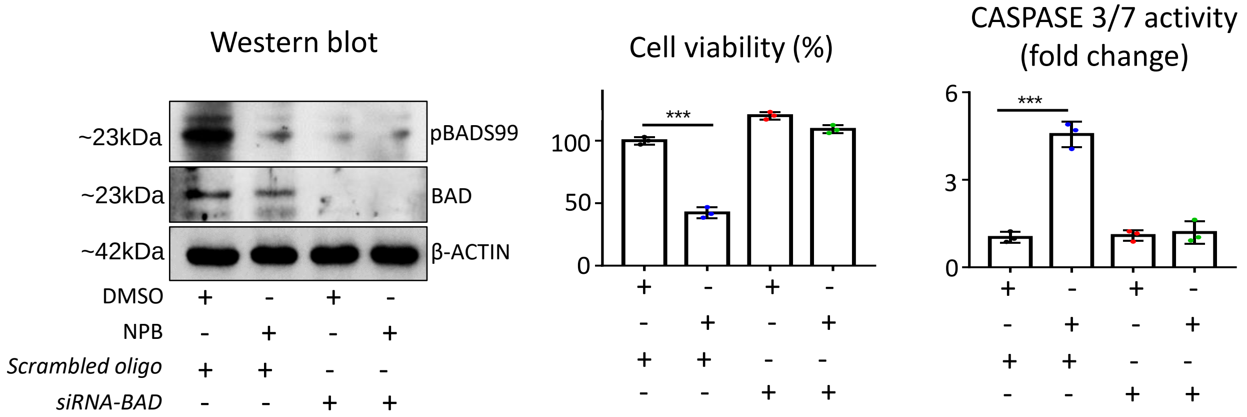

B. Combination efficacy of NPB and PARPis in CAOV2/CAOV2-*siRNA BAD* cells

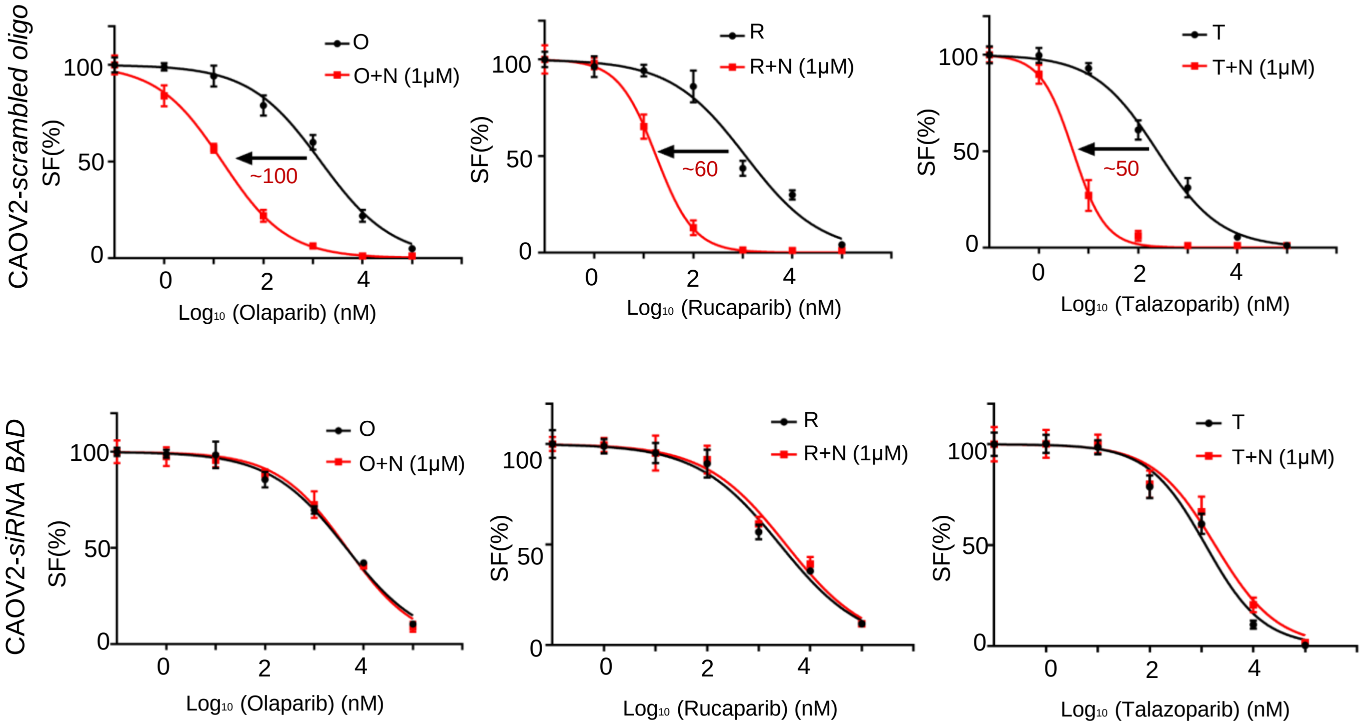

Supplementary Figure 3.

(A) *siRNA*-mediated depletion of BAD expression abrogated the effect of NPB in CAOV2 cells. WB analysis was used to assess the levels of pBADS99 and BAD protein in CAOV2 cells after treatment with 10μM NPB. Depletion of BAD expression was achieved using transient transfection of *siRNA* directed to the BAD transcript as previously described (1). Soluble whole-cell extracts were run on an SDS/PAGE gel and were immunoblotted as described in Materials and Methods. β-ACTIN was used as input control. CASPASE 3/7 activity and cell viability were evaluated using the ApoTox-Glo Triplex Assay Kit.

(B) Dose-response curves for CAOV2 cells and CAOV2 cells with *siRNA* mediated depletion of BAD treated with the indicated concentration of PARP inhibitors (PARPis, Olaparib=OLA, Rucaparib=RUC, Talazoparib=TAL) with or without 1uM NPB (N) in a total cell number assay. Survival fraction (SF) was assessed. Arrow indicates fold reduction in respective PARP inhibitor IC_{50} in the presence of NPB (n=3).

Data are represented as mean ± SD.

Supplementary Figure 4.

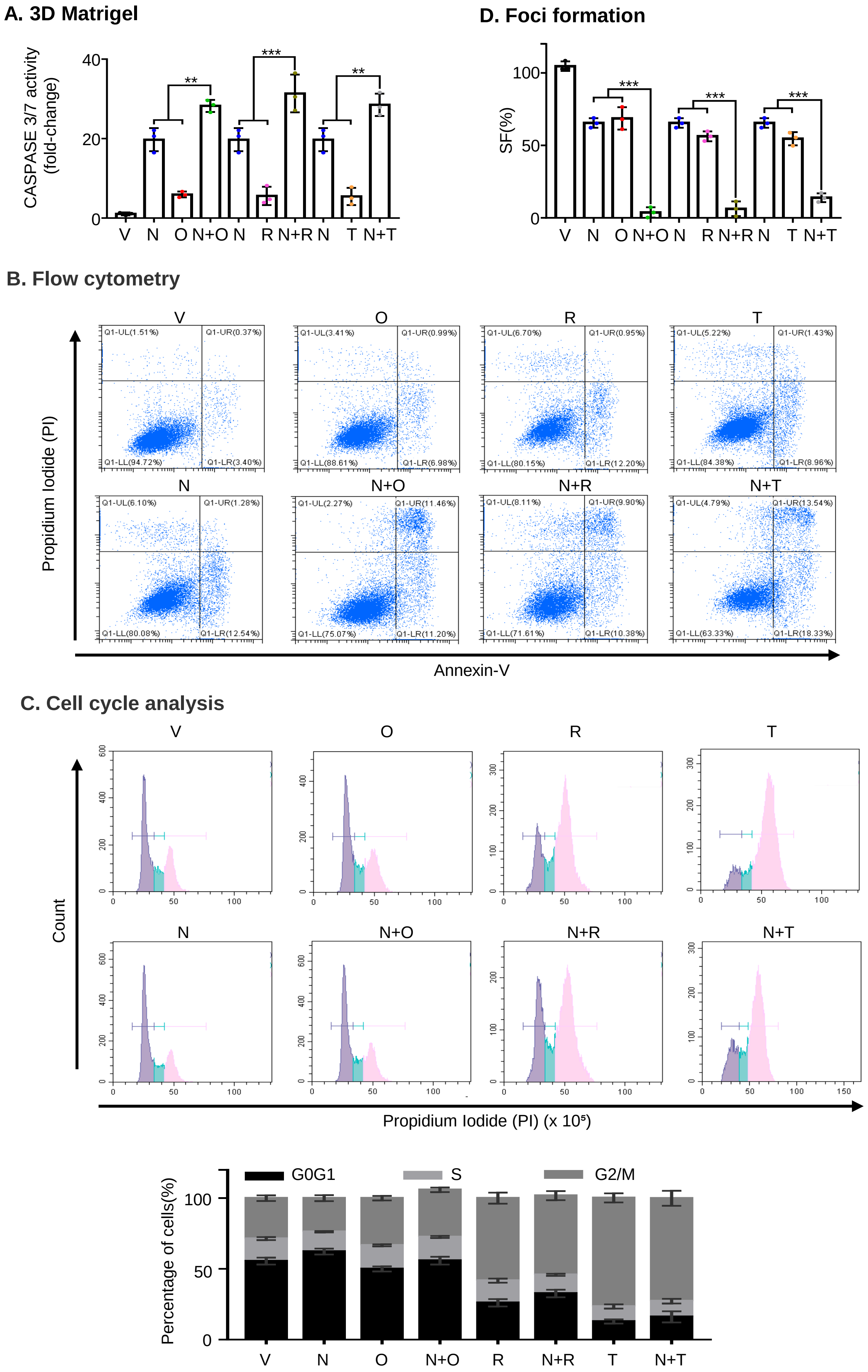

Supplementary Figure 4.

(A) CASPASE 3/7 activity was evaluated using the ApoTox-Glo Triplex Assay Kit in CAOV2 cells treated with NPB (N), PARP inhibitors (Olaparib=O, Rucaparib=R, Talazoparib=T) alone or in combination with the indicated anticancer agent concentrations for 14 days in 3D Matrigel culture (n=3).

(B) Representative flow cytometry plots using Annexin V-FITC/PI staining for apoptotic cell death of CAOV2 cells measured after treatment with NPB (N), Olaparib (O), Rucaparib (R), and Talazoparib (T) or combinations using flow cytometry analysis at 72 hours as described in materials and methods (n=3).

(C) Representative flow cytometry plots using PI staining for DNA of CAOV2 cells measured after treatment with NPB (N), Olaparib (O), Rucaparib (R), and Talazoparib (T) or combinations using flow cytometry analysis at 72 hours as described in materials and methods. Below: Sub-population of cells in G1, S, and G2-M phase after treatment by NPB (N), Olaparib (O), Rucaparib (R), and Talazoparib (T) or combinations (n=3).

(D) CAOV2 cells in foci formation assays were treated with NPB (N), Olaparib (O), Rucaparib (R), and Talazoparib (T) or combinations and at assay termination was stained with 0.2% crystal violet for 20 mins. To quantify, crystal violet stained colonies were resuspended in Isopropanol. The absorbance of soluble crystal violet (n=3) was detected at 570 nm using a microplate reader (Tecan Spark®, Switzerland). Percentage change in absorbance plotted as survival fraction (SF, %) of CAOV2 cells after treatment.

Columns are mean of triplicate experiments; bars, ±SD. *p < 0.05, ** p <0.01, *** p <0.001

Supplementary Figure 5.

A. Western blot analysis and quantification

B. IF quantification (CAOV2)

C. IF quantification (OVCAR433)

D. Co-immunoprecipitation of BAD with BCL-2, BCL-XL or BCL-W in CAOV2 cells

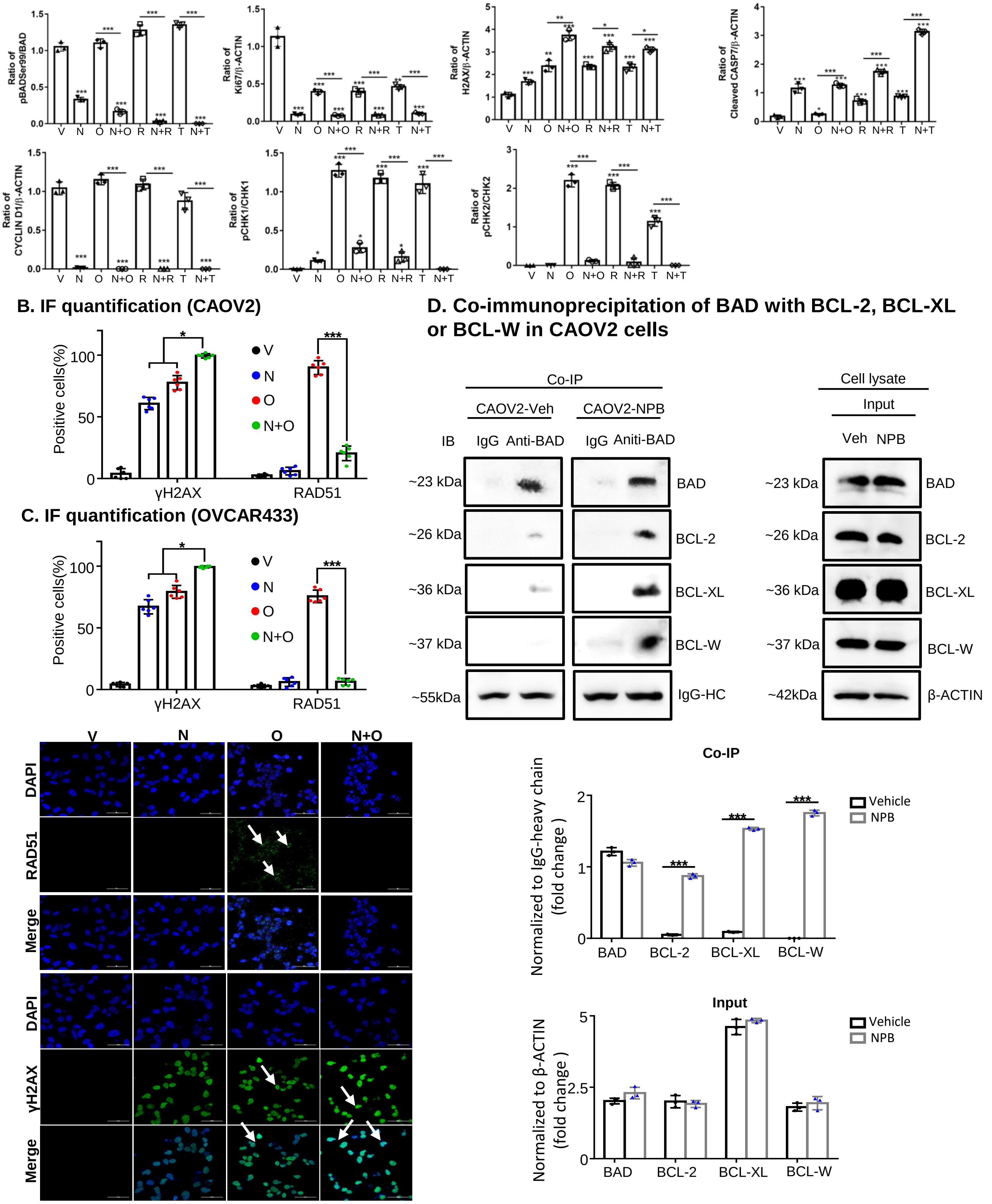

Supplementary Figure 5.

E. Caspase-dependent DNA damage

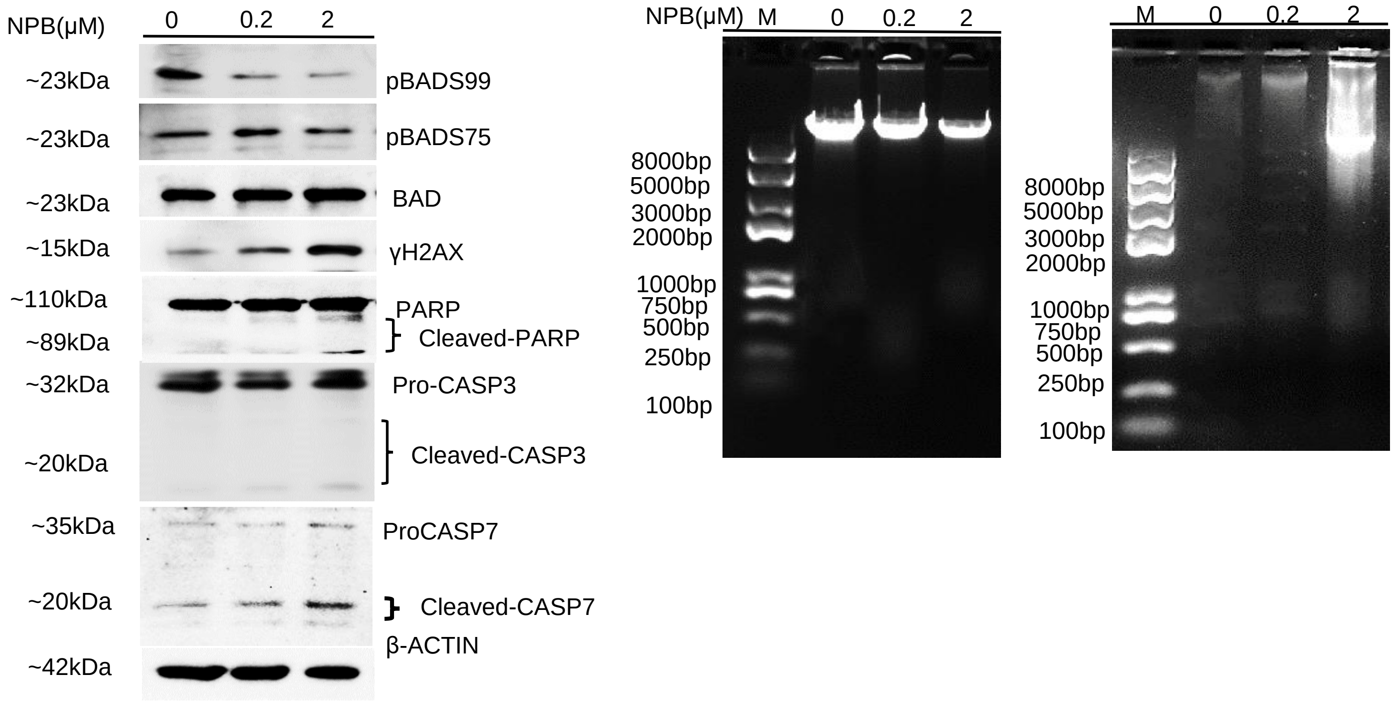

F. IF analysis

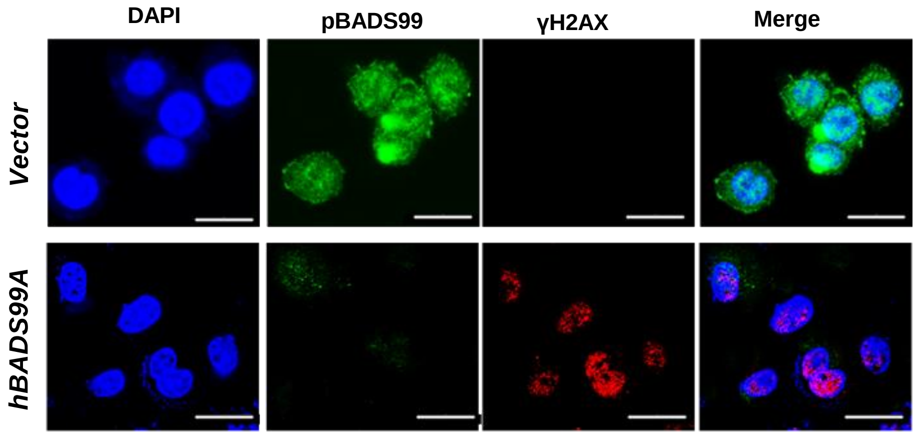

G. Homologous Recombination (HR) reporter assay

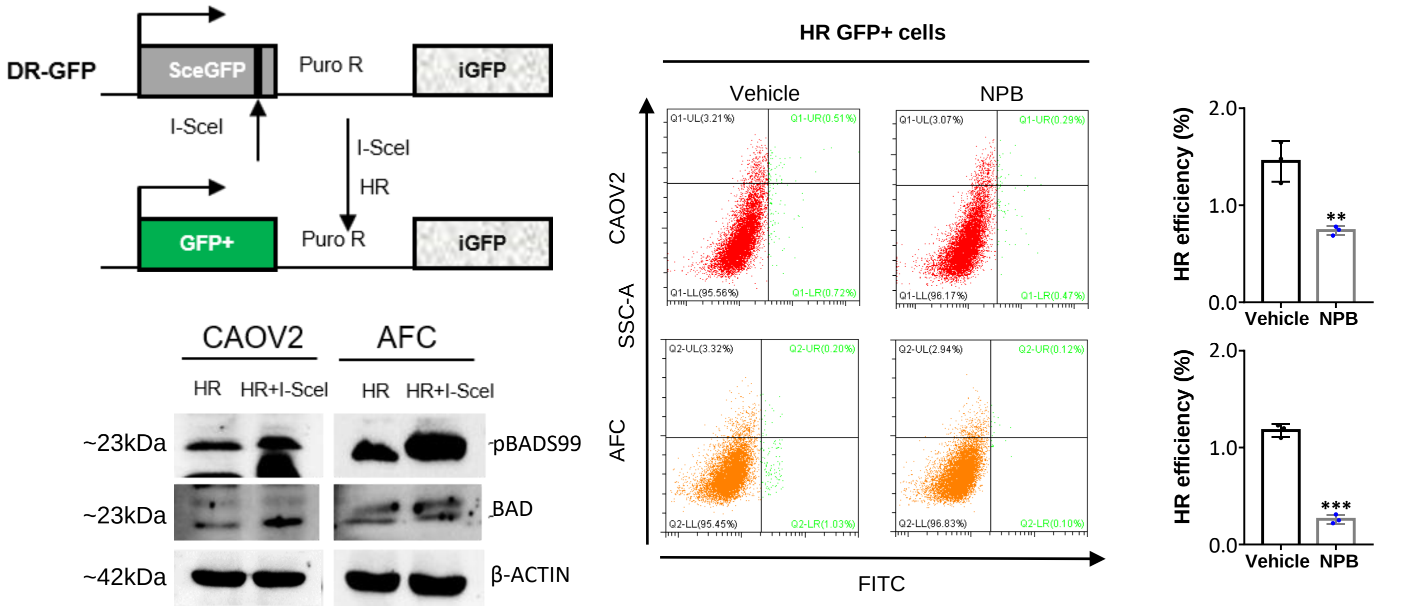

Supplementary Figure 5.

(A) Densitometry analysis of western blot data is represented in Figure 3E. Densitometry analysis of protein blots was determined using ImageJ software (https://imagej.nih.gov/ij/). Statistical changes were assessed by using an unpaired *two-tailed Student's t-test* ($P<0.05$ was considered significant), GraphPad Prism.

(B) Immunofluorescent (IF) semi-quantification of γH2AX and RAD51 foci positive CAOV2 cells treated with vehicle (V), NPB (N), Olaparib (O), or a combination (N+O) for 16 hours are shown. Cells with more than 5 RAD51 foci per nucleus were counted as RAD51 positive cells. 100 cells were analyzed in 2-3 separate fields for each sample (n=3).

(C) OVCAR433 cells were treated with vehicle (V), NPB (N), Olaparib (O), or a combination (N+O) for 16 hours and stained for γH2AX and RAD51 foci formation. Top: Immunofluorescence (IF) semi-quantification of γH2AX and RAD51 foci positive OVCAR433 cells are shown. Cells with more than 5 RAD51 foci per nucleus were counted as RAD51 positive cells. 100 cells were analyzed in 2-3 separate fields for each sample (n=3). Bottom: Representative images of γH2AX and RAD51 foci formation are taken by confocal microscopy. White arrows indicated positive staining. Scale bars, 50µm.

(D) Effect of NPB treatment on co-immunoprecipitation (co-IP) of BAD and BCL-2, BCL-XL, or BCL-W in CAOV2 cells. Lysate lane: whole cell lysate of vehicle (Veh) and NPB treated CAOV2 cells; Iso-IgG lane: co-IP using Iso-IgG-conjugated beads; co-IP BAD lane: IP using BAD antibody-conjugated beads. The heavy chain is denoted as HC. Immunoblotting is denoted as IB.

(E) Western blot analysis was used to assess the level of various protein markers and protein activities in CAOV2 cells after treatment with NPB (N) at the indicated concentrations. Soluble whole-cell extracts were run on SDS-PAGE and immunoblotted as described in materials and methods. β-ACTIN (ACTB) was used as input control for cell lysate. Genomic

DNA and apoptotic DNA fragments were isolated from NPB-treated CAOV2 cells as described in the supplementary text and were run on a 2% TAE agarose gel.

(F) Forced expression of Flag-hBADS99A in CAOV2 cells exhibited no p-BADS99 immunoreactive signal and induced γH2AX expression as confirmed by immunofluorescent (IF) analysis, Scale bar, 20 μm.

(G) The *HR-GFP reporter* consists of two copies of mutant *GFP* genes. Cells were transfected with I-SceI expressing vector to induce DSB and which may be repaired by homologous recombination (HR) using iGFP as a template and restoring a functional *GFP* gene. The percentage of GFP-positive (GFP+) cells was measured using flow cytometry as an indicator of HR efficiency. Cells with DSBs introduced by I-SceI endonuclease HR exhibit increased levels of pBADS99 in EOC cells as determined by western blot analysis. A reduction is observed in GFP-positive cells after NPB treatment in BRCA wild-type and mutant EOC cells (n = 3).

Columns are mean of triplicate experiments; bars, ±SD. *p < 0.05, ** p <0.01, *** p <0.001

Supplementary Figure 6.

A. Clinical information of PDOs

Patient ID	Tissue type for PDO	Pathological subtype	Age at diagnosis (years)	FIGO stage	Surgery	Chemotherapy	Metastasis	Family history
**PDO1**	Metastatic ascites	HGSOC	54	FIGO IV	yes	TP (8 cycles)	peritoneal liver	N
**PDO2**	Primary debulking	HGSOC	43	FIGO III	yes	NY	peritoneal	N
**PDO3**	Interval debulking	HGSOC	65	FIGO III	yes	Adjuvant chemotherapy TP (3 cycles)	peritoneal	N
**PDO4**	Primary debulking	HGSOC	56	FIGO III	yes	NY	peritoneal	N

HGSOC: high grade serous ovarian cancer; FIGO: The International Federation of Gynecology and Obstetrics.
TP: Paclitaxel and platinum
NY: Not yet; N:No

B. 2D culture: patient derived cell morphology

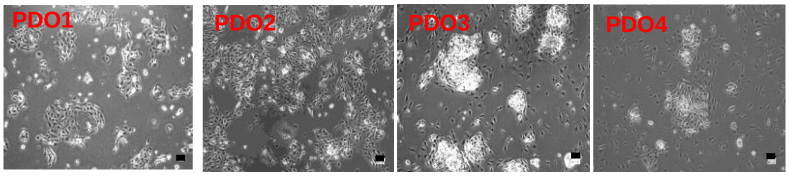

C. Western blot

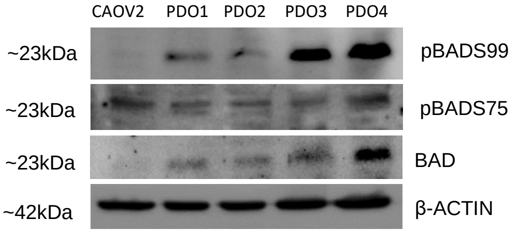

D. 2D-Matrigel: NPB and PARP inhibitor combination in AFC cells (PDO1)

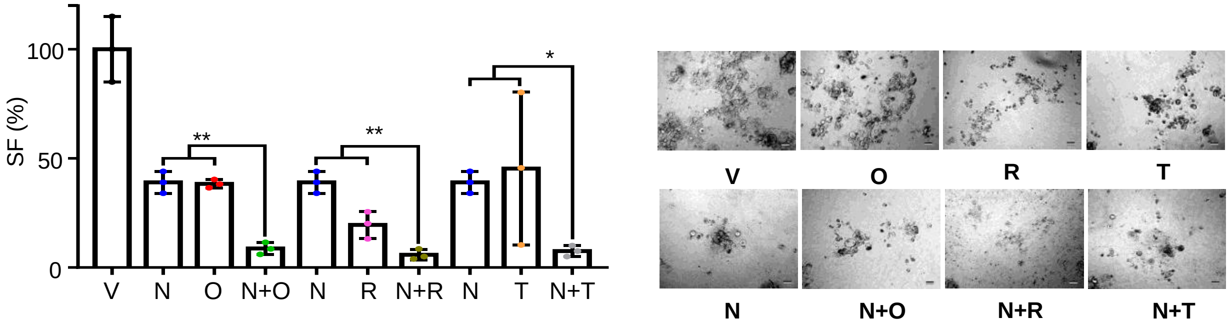

E. 3D Matrigel: live/dead PDO-1 cells

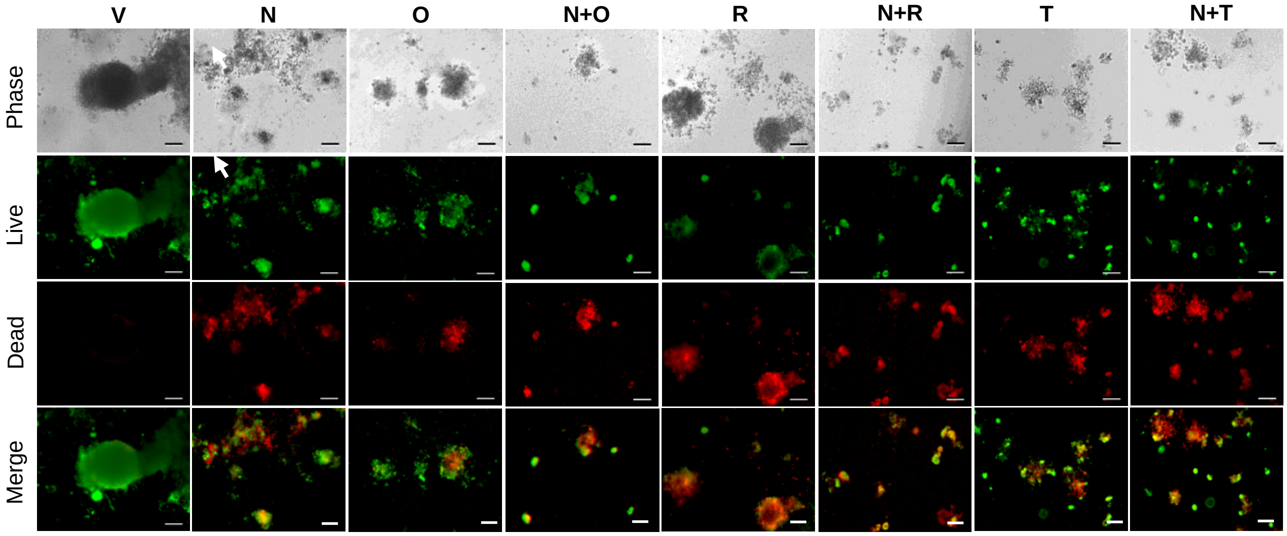

Supplementary Figure 6.

F. Western blot quantification (PDO1)

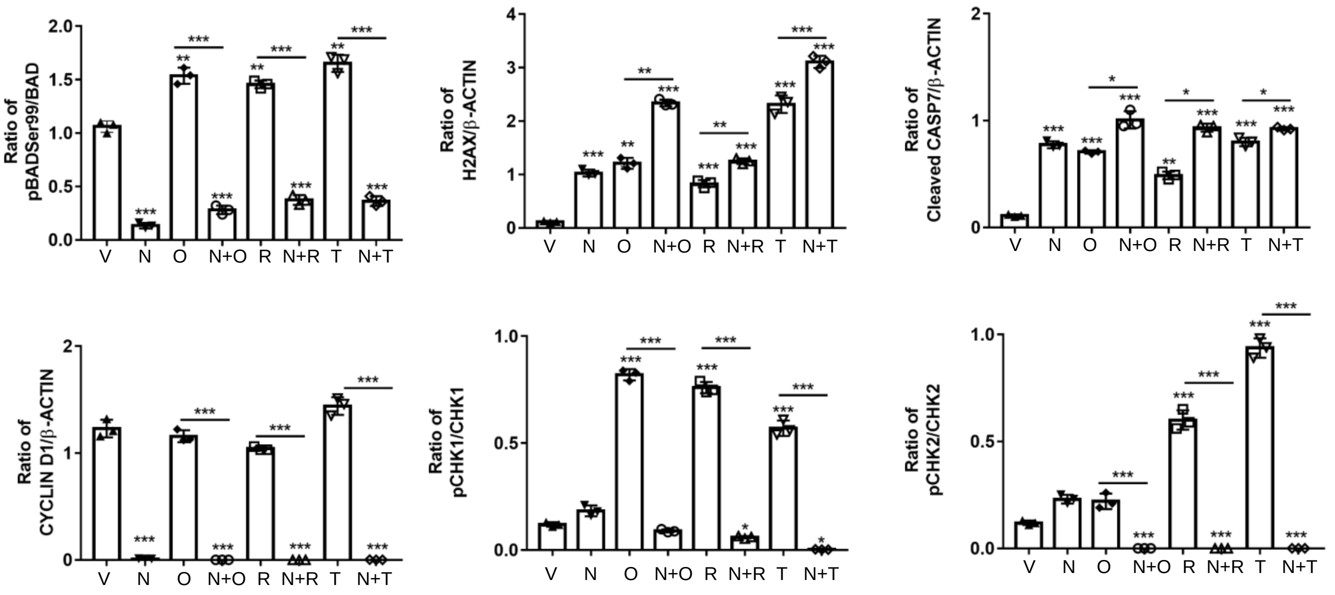

G. PDO1/PDO1-*siRNA* BAD

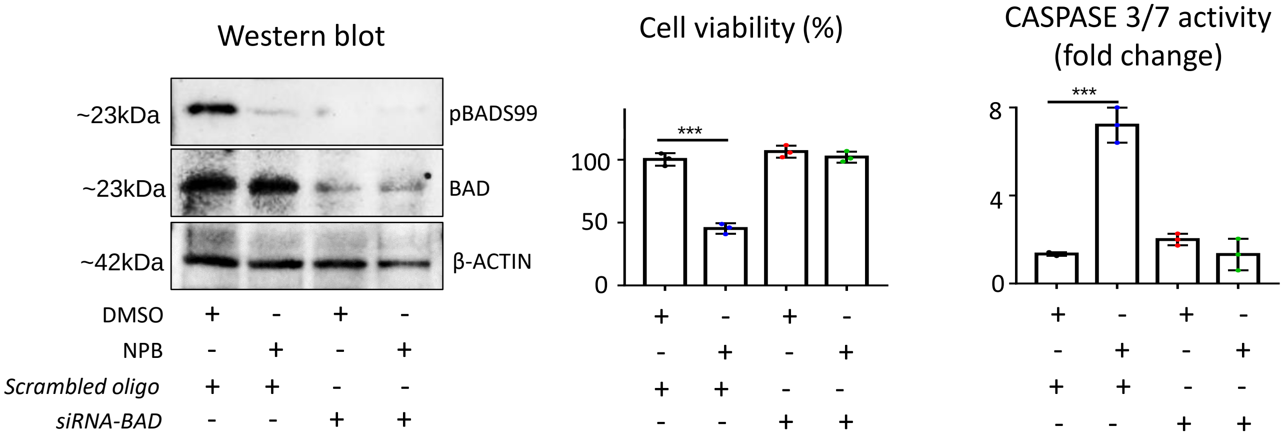

Supplementary Figure 6.

(A) Clinical information of patient-derived EOC organoid (PDO) 1-4 patients.

(B) Patient-derived EOC organoid (PDO) cell morphology in 2D culture.

(C) Western blot analysis was used to assess the levels of pBADS75, pBADS99, and BAD protein in patient-derived EOC organoid (PDO) cells. Soluble whole-cell extracts were run on an SDS-PAGE and immunoblotted as described in materials and methods. β-ACTIN was used as input control for cell lysate. The sizes of detected protein bands in kDa are shown on the left side.

(D) AFC (patient-derived EOC organoid (PDO) 1) cells treated with NPB (N), PARP inhibitors (Olaparib=O, Rucaparib=R, Talazoparib=T), or a combination for one week in 2D Matrigel culture. Cell viability represented as survival fraction (SF) was evaluated using Alamarblue assay (n=3).

(E) Microscopic visualization of calcein-AM (green) stained colonies (live) and PI (red) stained cell debris (dead) generated by AFC (patient-derived EOC organoid (PDO) 1) cells cultured in 3D Matrigel after exposure of pregrown colonies to NPB (N), PARP inhibitors (Olaparib=O, Rucaparib=R, Talazoparib=T) and combination (n=3). White arrows indicate positive staining. Scale bars, 100µm.

(F) Densitometric analysis of western blots in Figure 4C. AFC (patient-derived EOC organoid (PDO) 1) cells were treated with NPB (N) and PARP inhibitors (Olaparib=O, Rucaparib=R, Talazoparib=T). Densitometric analysis of protein blots was determined using ImageJ software (https://imagej.nih.gov/ij/). Statistical changes were assessed by using an unpaired two-tailed Student’s t test ($P<0.05$ was considered as significant), GraphPad Prism.

(G) siRNA-mediated depletion of BAD expression abrogates the effect of NPB in patient-derived EOC organoids (PDO) 1: WB analysis was used to assess the levels of pBADS99 and BAD protein in PDO1 after treatment with NPB. Depletion of BAD expression was achieved using

transient transfection of siRNA directed to the BAD transcript as previously described (Pandey et al, 2018, PNAS (Figure 6A&B)). Organoids were harvested after depolymerization of Matrigel using Cultrex® organoid harvesting solution (R&D systems, US) and the organoid-pellet was resuspended in RIPA buffer plus protease inhibitors. Extracts were run on an SDS-PAGE and immunoblotted as described in materials and methods. β-ACTIN was used as input control. Cell viability was evaluated using the ApoTox-Glo Triplex Assay Kit. Caspase 3/7 activities were evaluated using the ApoTox-Glo Triplex Assay Kit.

Columns are mean of triplicate experiments; bars, ±SD. *$p < 0.05$, ** $p < 0.01$, *** $p < 0.001$

Supplementary Figure 7.

A. Combined NPB and PARPi treatment in PDOs

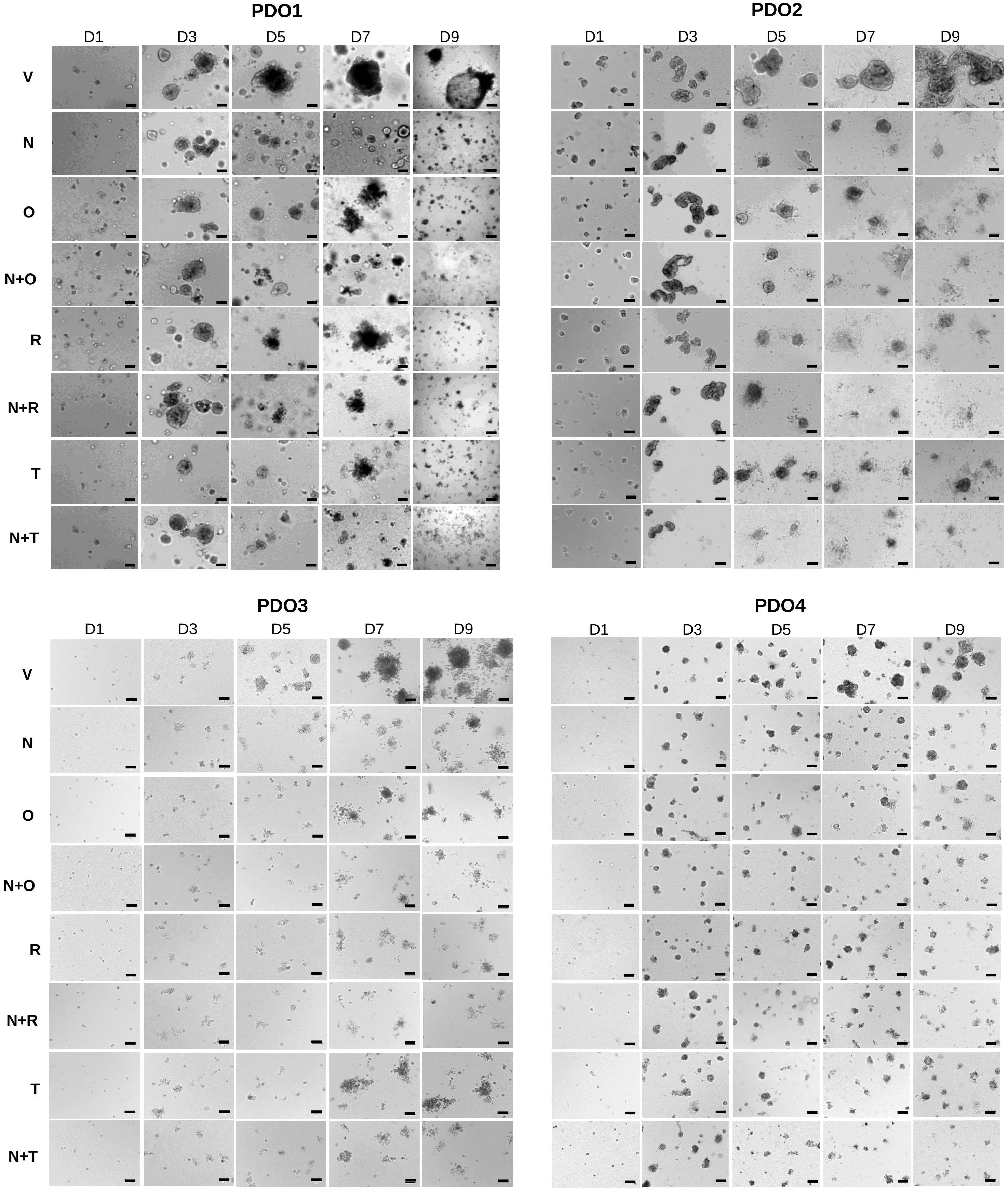

Supplementary Figure 7.

B. PDO growth curve

PDO1

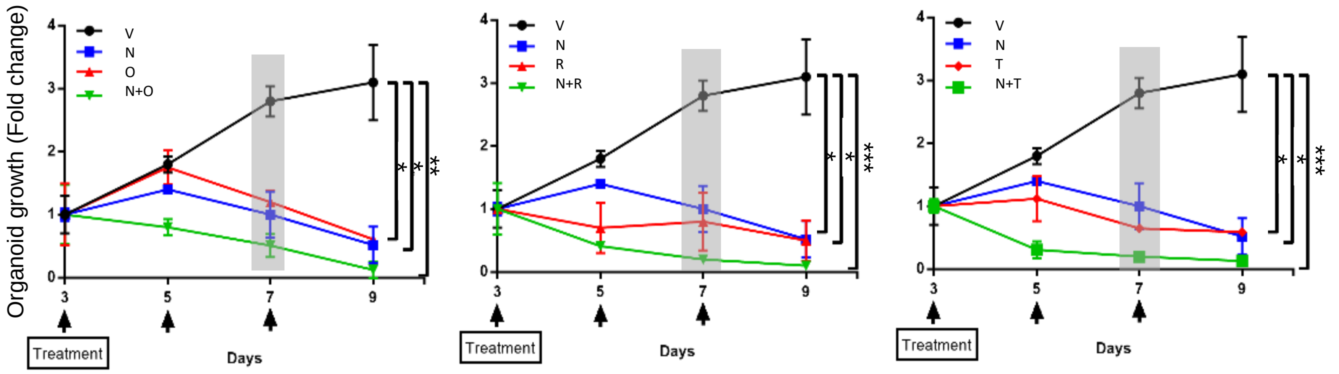

PDO2

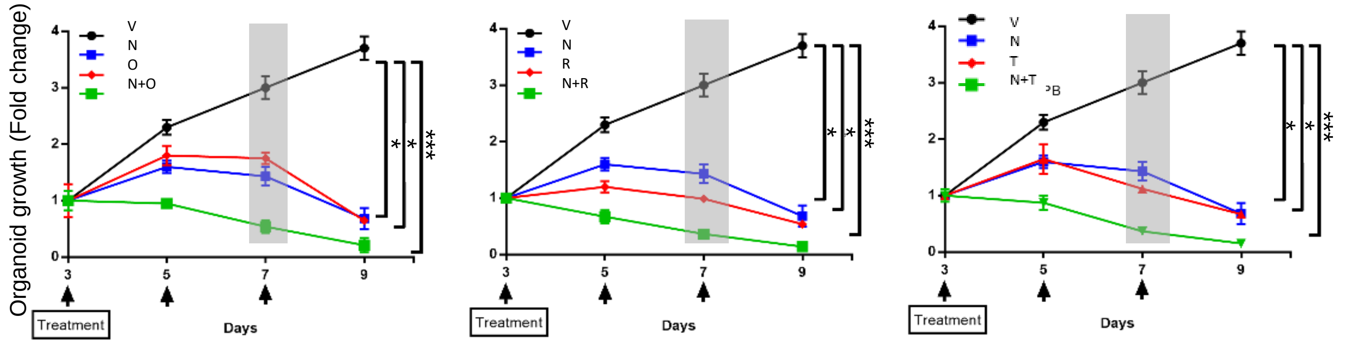

PDO3

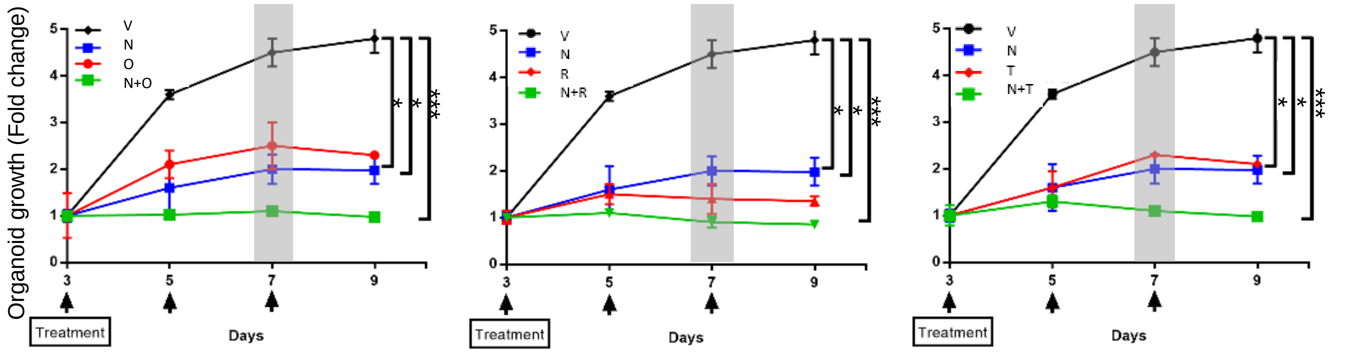

PDO4

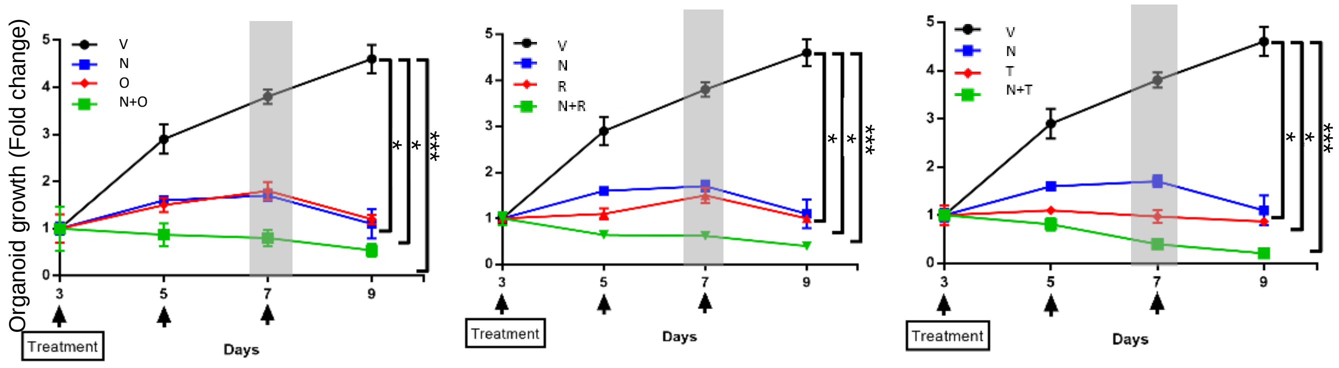

Supplementary Figure 7.

(A) Three days post-embedding, pre-grown patient-derived EOC organoids (PDOs) were treated with NPB (N), PARP inhibitors (PARPis, Olaparib=O, Rucaparib=R, Talazoparib=T), or a combination every 2 days. Images of the PDOs were captured every 2 days. Organoids with diameters >100μm were counted over the 9-day culture.

(B) The growth curve of patient-derived EOC organoid (PDO) was depicted by counting organoids with diameters >100μm in number during 9-day experimental culture.

Points are mean of triplicate experiments; bars, ±SD. *p < 0.05, ** p <0.01, *** p <0.001

Supplementary Figure 8.

A. Scheme of xenograft protocol

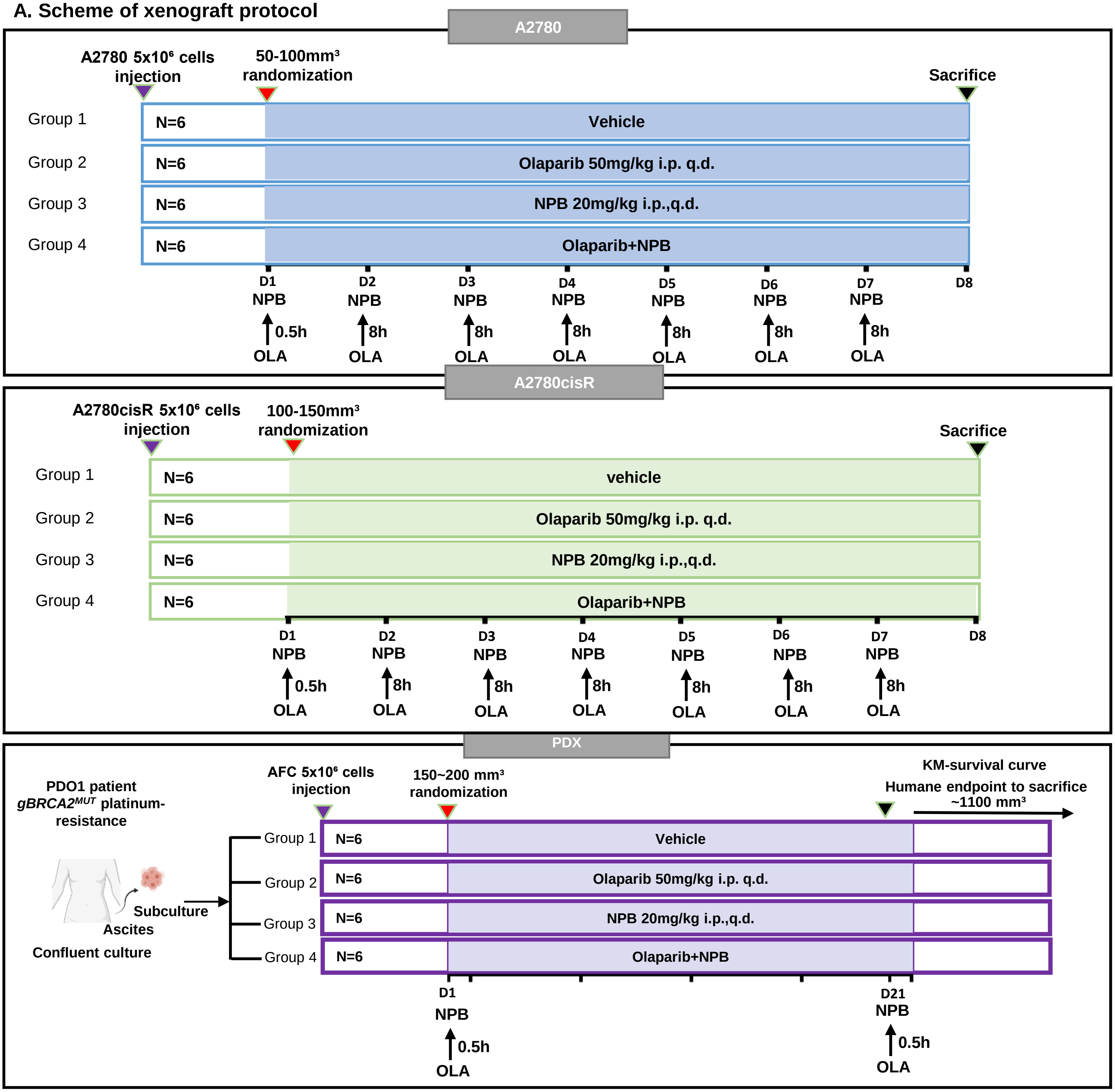

B. Representative resected xenografts

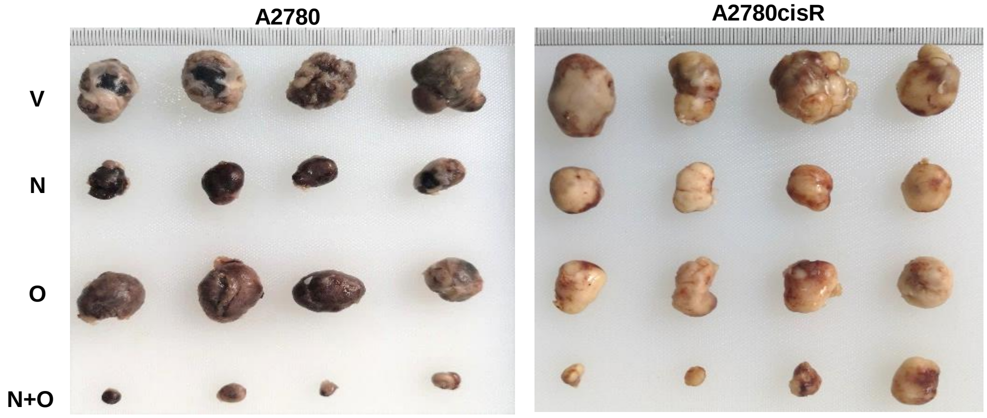

C. Immunoreactivities in CRISPR-CAS9 mediated deletion of BAD in A2780 cells

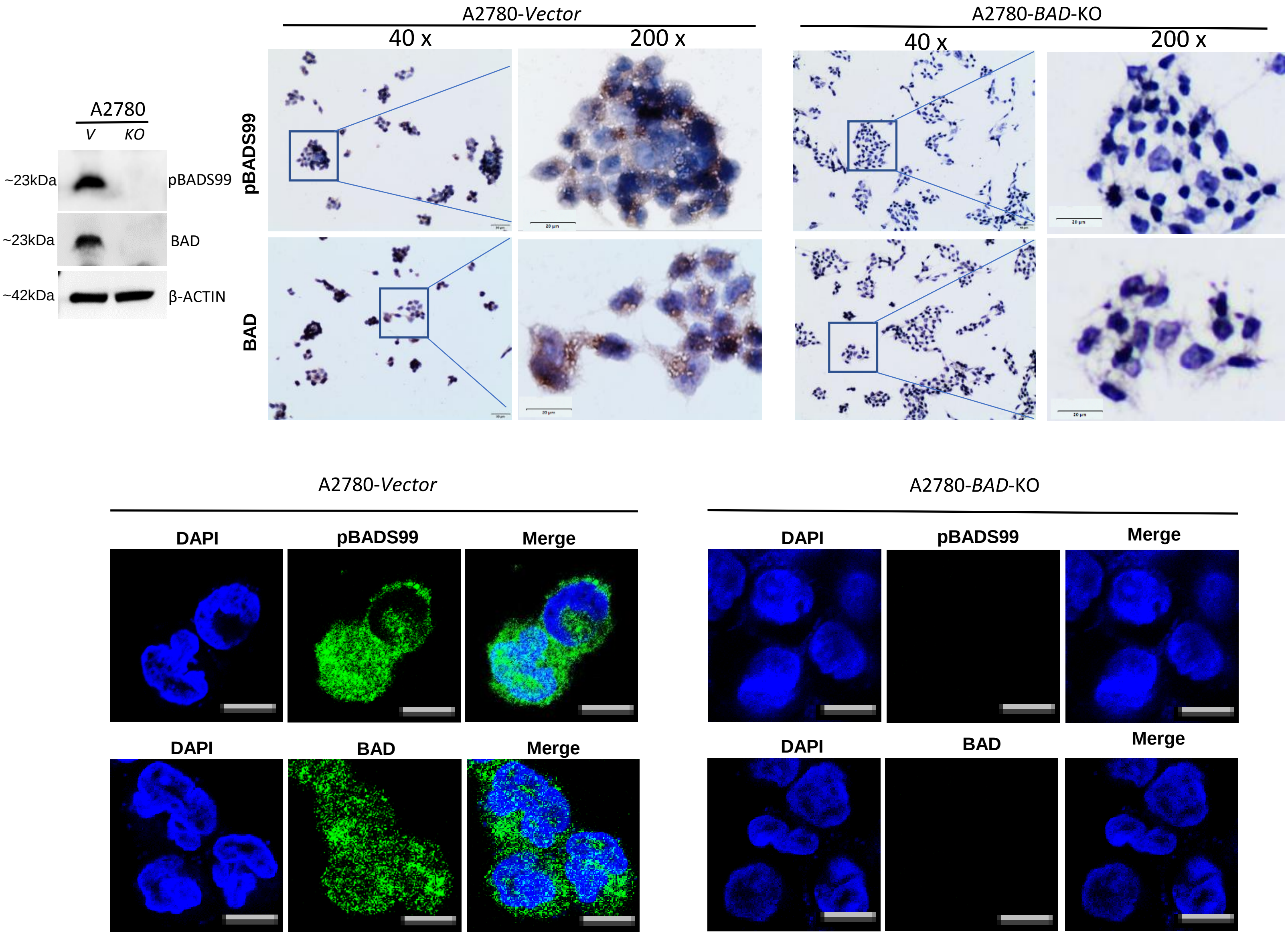

Supplementary Figure 8.

D. Immunohistochemistry

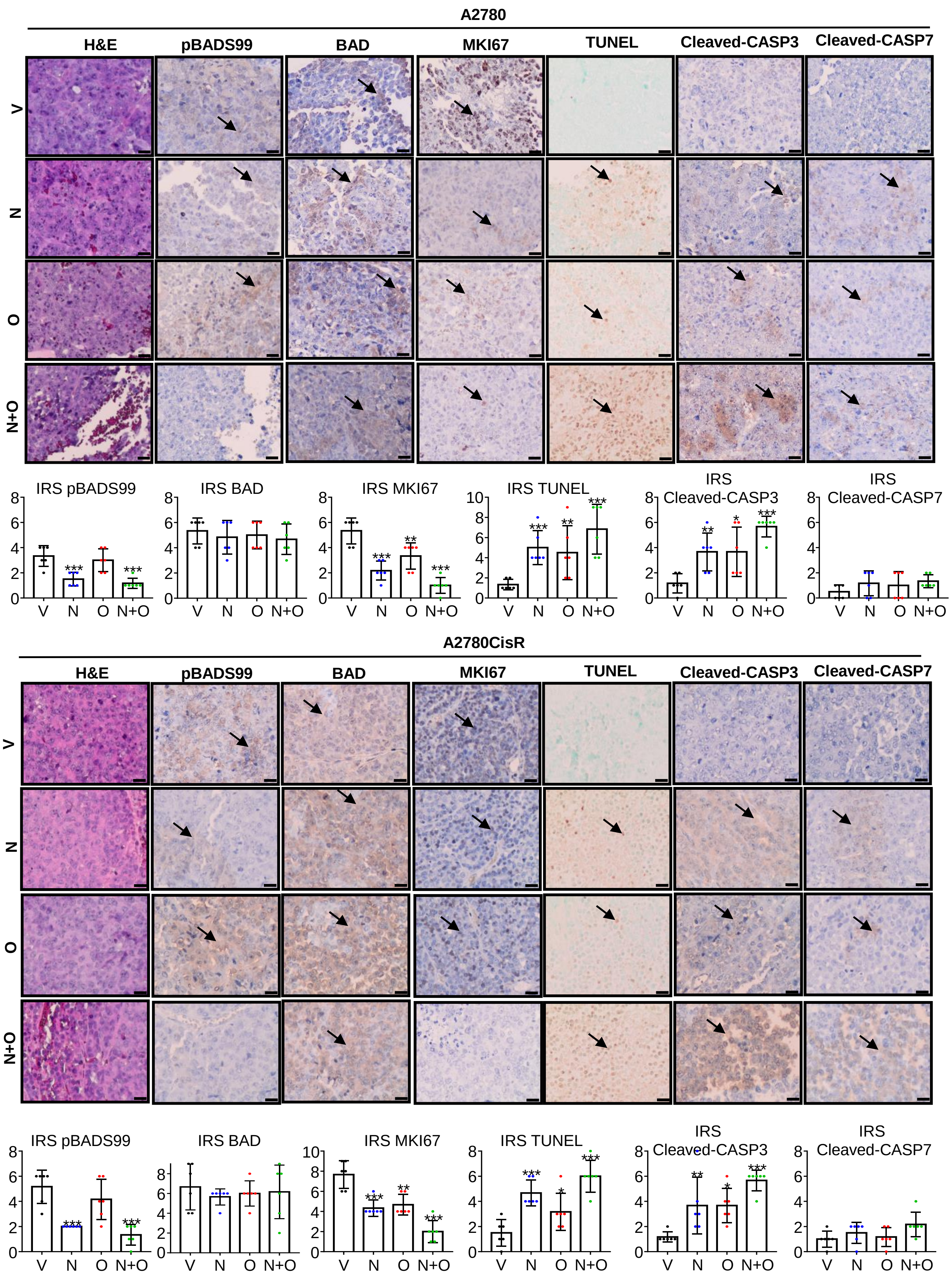

Supplementary Figure 8.

(A) Scheme for xenograft treatment: A2780, A2780CisR (cisplatin-resistant), and AFC (patient-derived EOC organoid (PDO) 1) cells were injected subcutaneously into the flank of the BALB/c athymic mice, respectively. In each cell group, the mice were randomized into 4 indicated treatment groups (n = 6) after xenograft generation and treated daily with vehicle, 20 mg/kg NPB, 50 mg/kg Olaparib (OLA), or a combination of 1 week (A2780 and A2780CisR xenograft) or 3 weeks (AFC xenograft). Xenograft volumes and mice weights were measured daily. Xenograft studies were performed according to the Animal Research: Reporting In Vivo Experiments (ARRIVE) 2.0 guidelines.

(B) Mice were treated daily with vehicle, 20 mg/kg NPB, 50 mg/kg Olaparib (OLA), or combination. Shown images are representative of dissected xenografts from the indicated treatment groups.

(C) Representative images of BAD and pBADS99 immunoreactivities (western blot, immunocytochemistry, and immunofluorescence) in CRISPR-CAS9 mediated knockout (KO) of BAD in A2780 cells. Top right: In immunocytochemistry, 40x magnificent, Scale bar, 50μm and 200x magnificent, Scale bar, 20μm. Below: in immunofluorescence, 1000x magnificent, Scale bar, 20μm.

(D) Hematoxylin and eosin (HE), pBADS99, BAD, KI67, cleaved-CASP3/7, and TdT-mediated dUTP Nick-End Labeling (TUNEL) positivity was assessed in resected xenografts treated with vehicle (V), 20 mg/kg NPB (N), 50 mg/kg Olaparib (O) or combined NPB-Olaparib by i.p. injection using immunohistochemistry (IHC) as described in materials and methods. Quantification of immunoreactivity score (IRS) from the indicated treatment group is shown in the individual value plot (n=6). All images were captured at × 400 magnification. Black arrows indicate positive staining. Scale bars, 20μm. Black arrows indicate positive staining; Below: the average IRS of each marker in different groups are presented.

Columns and points are mean of triplicate experiments; bars, ±SD. *$p < 0.05$, ** $p < 0.01$, *** $p < 0.001$

Supplementary Figure 9.

In-Silico Genotoxicity Prediction for NPB

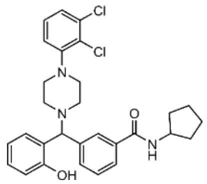

1. Outcome of GT1_BMUT Model：Negative

Positive Alerts	Deactivating Features	Unknown Fragments
**2**	4	**0**
**Alert ID 47: cH:c(:c)-N3**<br>**Alert ID 97:**<br>**c-N3(-C3H2)-C3H2**	**Fragment ID 320: c:(:c)-Cl Fragment ID**<br>**498: Cl-c1:cH:cH:cH:c:c1-Cl**<br>**Fragment ID 1144: c:cH:c(:cH:cH)-C2(-N3H(=O**<br>**Fragment ID 1906: cH:cH:c(-OH):c(-C3H):cH**	

There are 2 positive alerts and 4 deactivating features with the model. The QSAR calculated probability is 4.7%, which is lower than the model’s current classification threshold (50.0%) and within the gray zone (40.0% ~ 60.0%).

2. Outcome of Expert Rule Model: Negative

Positive Alerts	Deactivating Features	Unknown Fragments
**0**	0	**0**

There is no positive alert with the model. The QSAR calculated probability is 0.0%, which is lower than the model’s current classification threshold (50.0%) and not within the gray zone (40.0% ~ 60.0%). The outcome of GT1_BMUT model is negative, and the outcome of Expert Rule model is negative. To sum up, the prediction result of the compound is negative and belongs to a potential ICH M7 Class 5 compound.

Expert Review

The outcome of GT1_BMUT model is negative, and the outcome of Expert Rule model is negative. To sum up, the prediction result of the compound is negative and belongs to a potential ICH M7 Class 5 compound.

Supplementary Figure 9.

In Silico Genotoxicity Prediction for NPB

The genotoxicity predictions were evaluated by the statistical model GT1_BMUT and the expert rules model GT_EXPERT in CASE Ultra (version: v1.8.0.2) software, respectively. Meanwhile, the konsolidator database BMUT_KONSOLIDATOR_DB (ver 4.0) with experimental data has been used to facilitate expert analysis (ICAS, China).

Supplementary Figure 10.

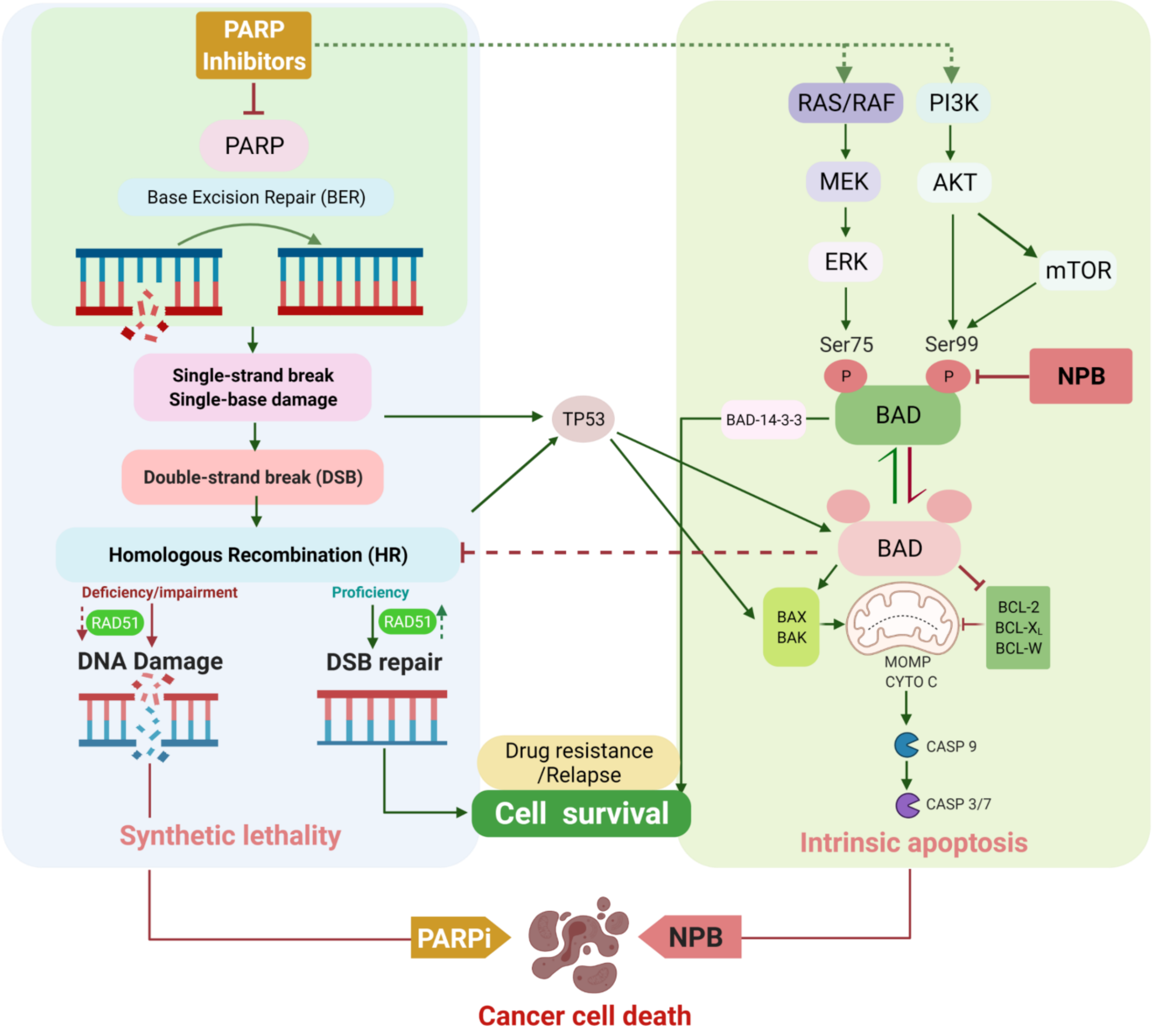

Supplementary Figure 10.

Interaction between the intrinsic apoptosis and DNA damaging processes in EOC provides a rational strategy for combination of PARP inhibitors and NPB.

Poly (ADP-Ribose) polymerase (PARP), as a major component of the DNA damage response (DDR), is recruited to initiate cellular responses to single-strand DNA breaks (SSB). PARP inhibition, therefore, results in cell death through synthetic lethality in EOC cells with homologous recombination (HR) deficiency (eg loss of BRCA1/2 function). PARP inhibition in EOC cells has been reported to stimulate the activation of the RAS/MEK/ERK and PI3K/AKT/mTOR signaling pathways (2-5). The RAS/MEK/ERK and PI3K/AKT/mTOR signaling cascades converge at BCL-2 associated death promoter (BAD) and participate in BAD phosphorylation at Serine (S)75 residue primarily through the p44/42 MAP kinase pathway (6) and at S99 residue primarily through activation of PI3K/AKT/mTOR (7, 8) to promote cancer cell survival; and which is one of the mechanisms of drug resistance and cancer relapse. Treatment of HR-proficient EOC with the BAD phosphorylation inhibitor NPB induced intrinsic apoptosis and attenuated HR efficiency. In response to DNA damage, TP53 upregulates BAD transcription. Dephosphorylated BAD also heterodimerizes with TP53 (9) or with BCL2, BCL2-XL, and BCL-W and sequesters BCL2, BCL2-XL, and BCL-W with consequent intrinsic apoptosis through disruption of mitochondrial membrane potential (10). The functional interaction between the intrinsic apoptosis and DNA damage processes in EOC implies that a combination of inhibition of both BAD phosphorylation and PARP may ameliorate outcomes in recurrent EOC.

Supplementary Figure 11. Uncropped western blot images.

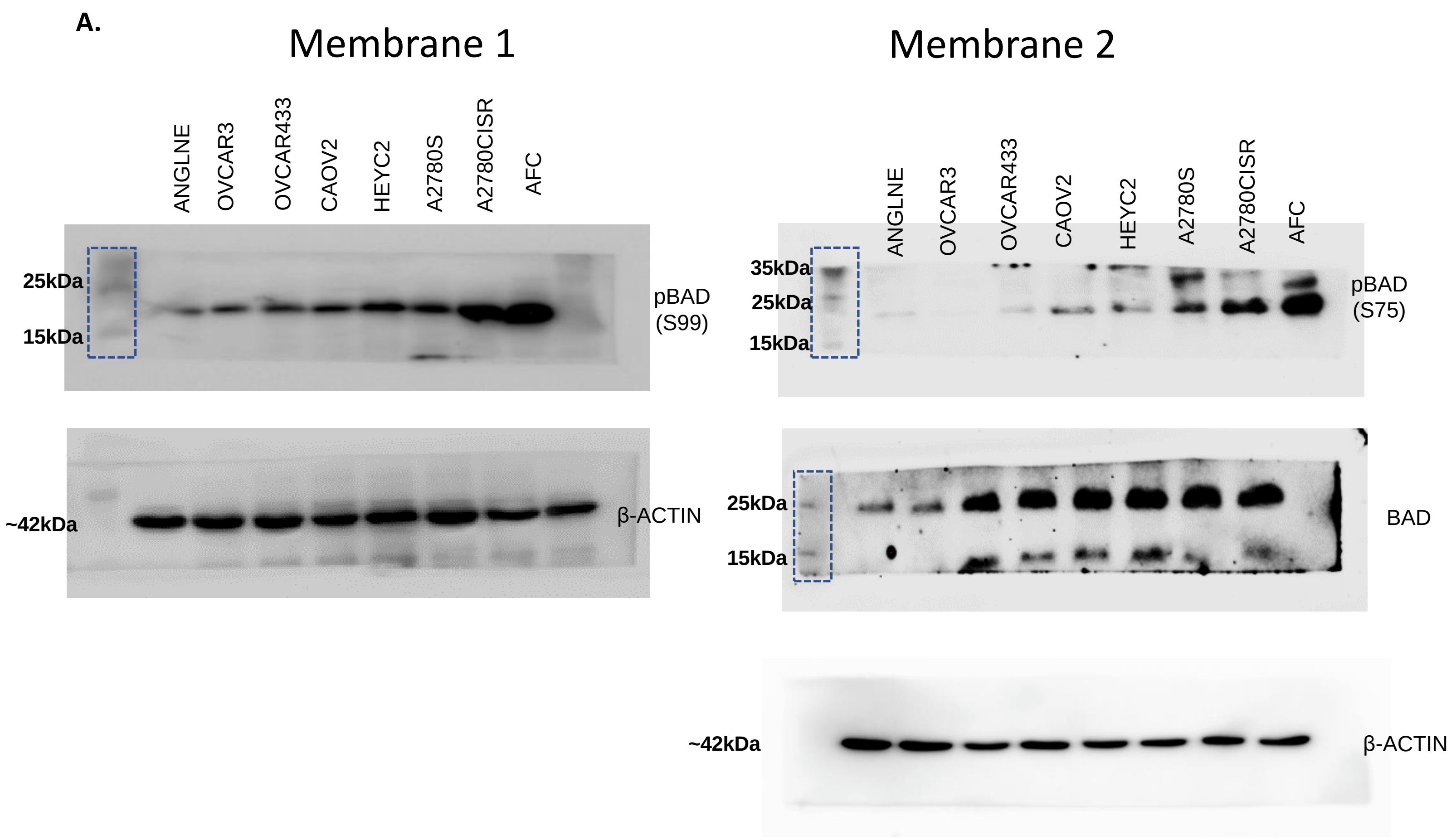

Supplementary Figure 11. Uncropped western blot images.

B.

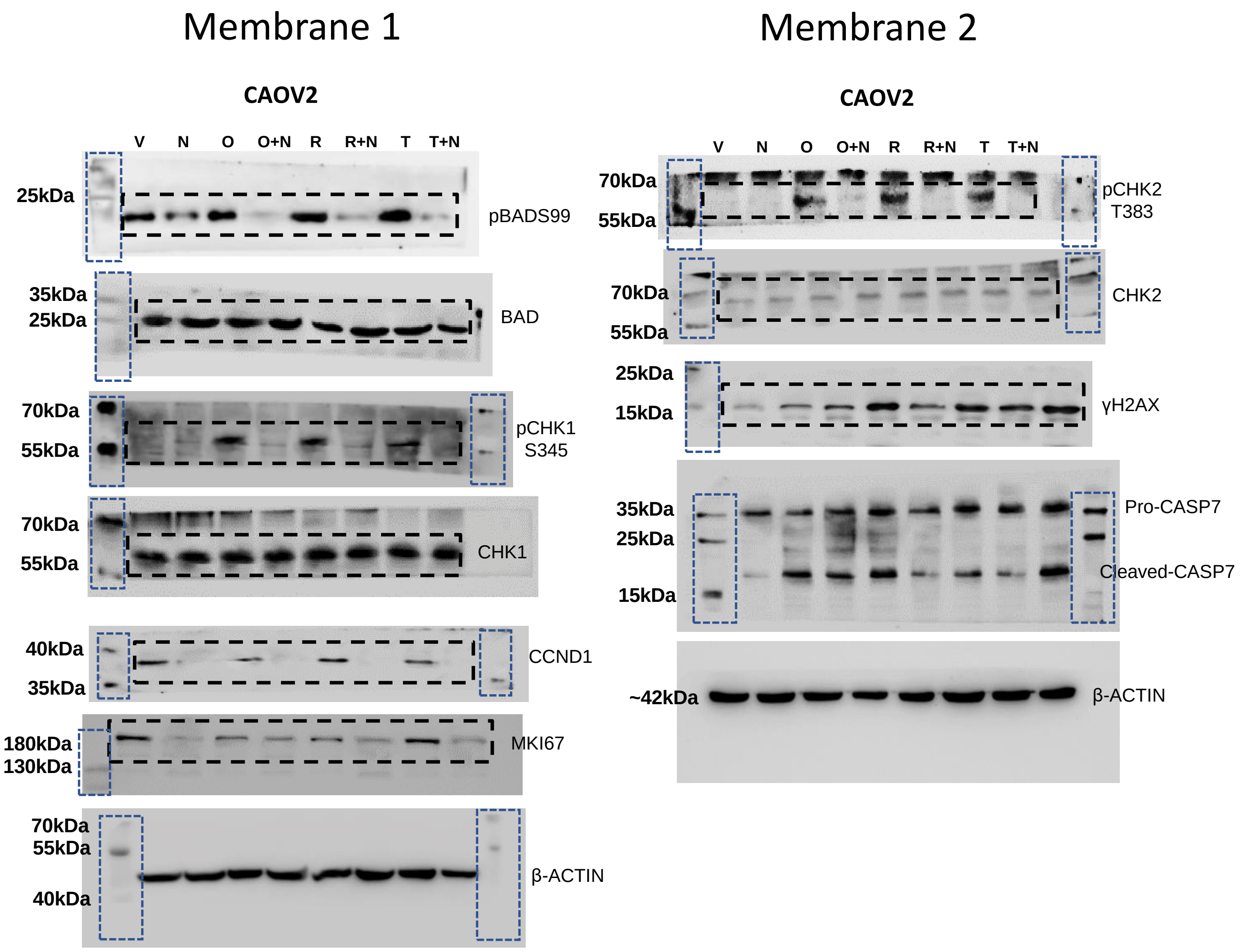

Supplementary Figure 11. Uncropped western blot images.

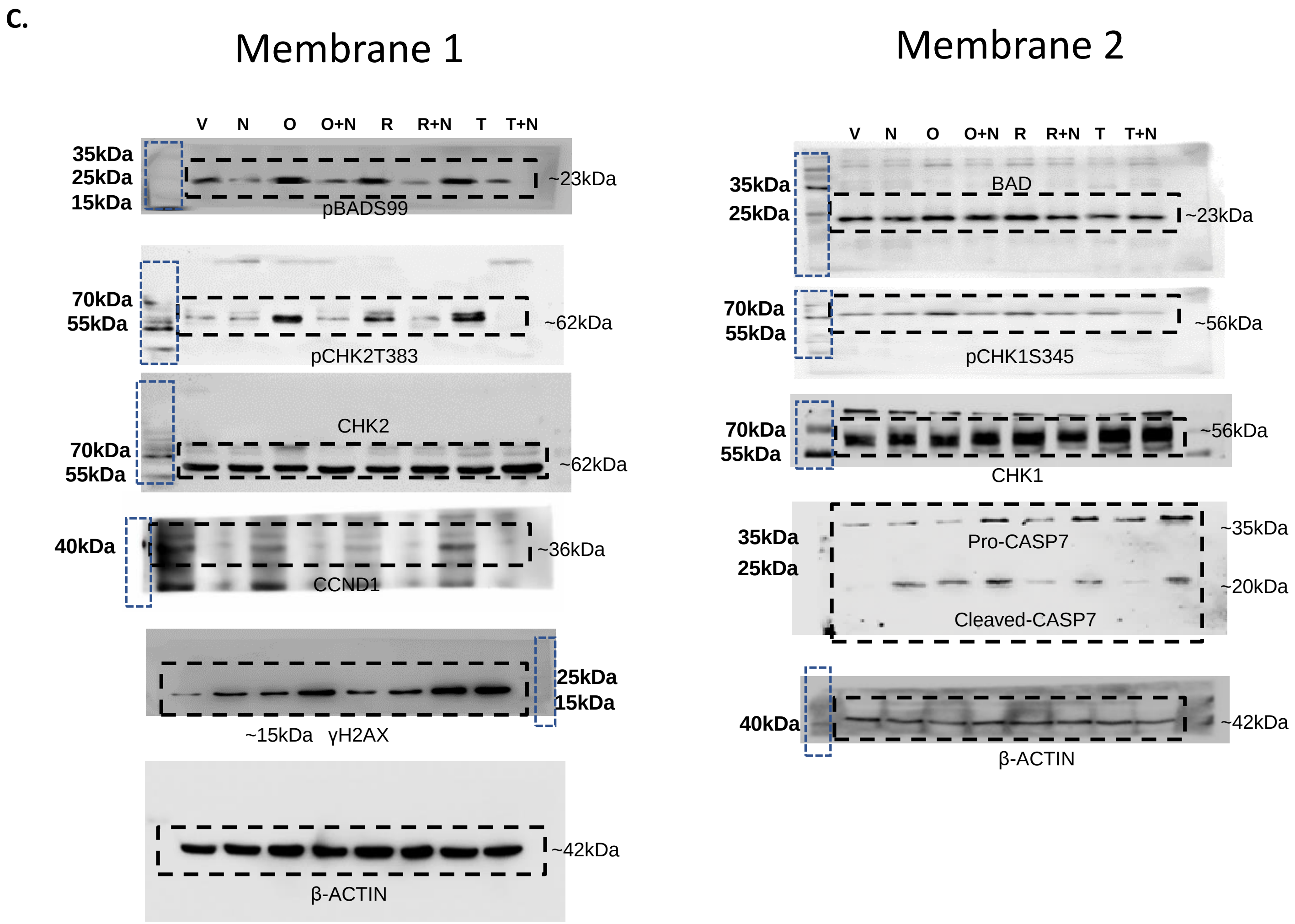

Supplementary Figure 11. Uncropped western blot images.

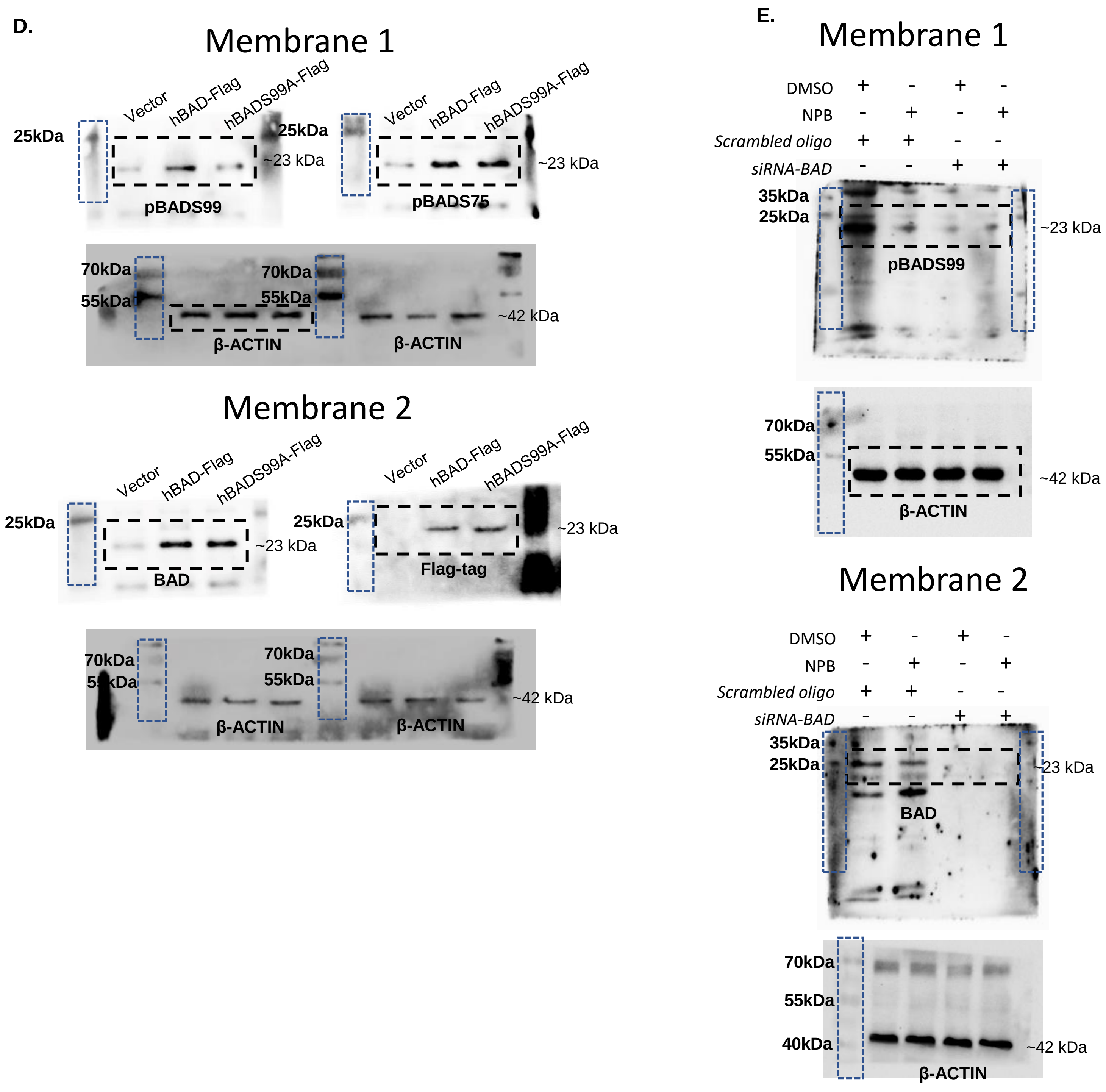

Supplementary Figure 11. Uncropped western blot images.

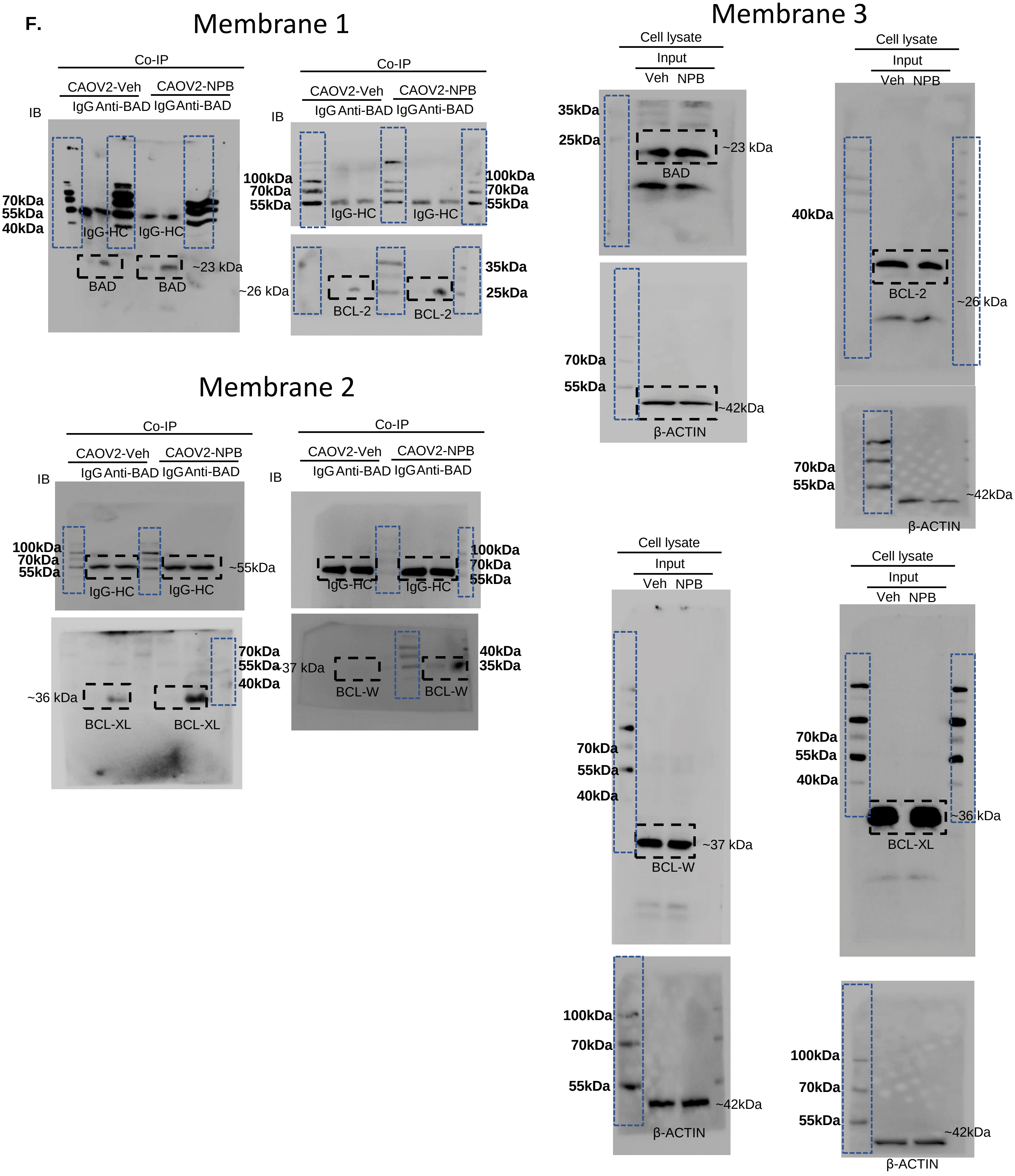

Supplementary Figure 11. Uncropped western blot images.

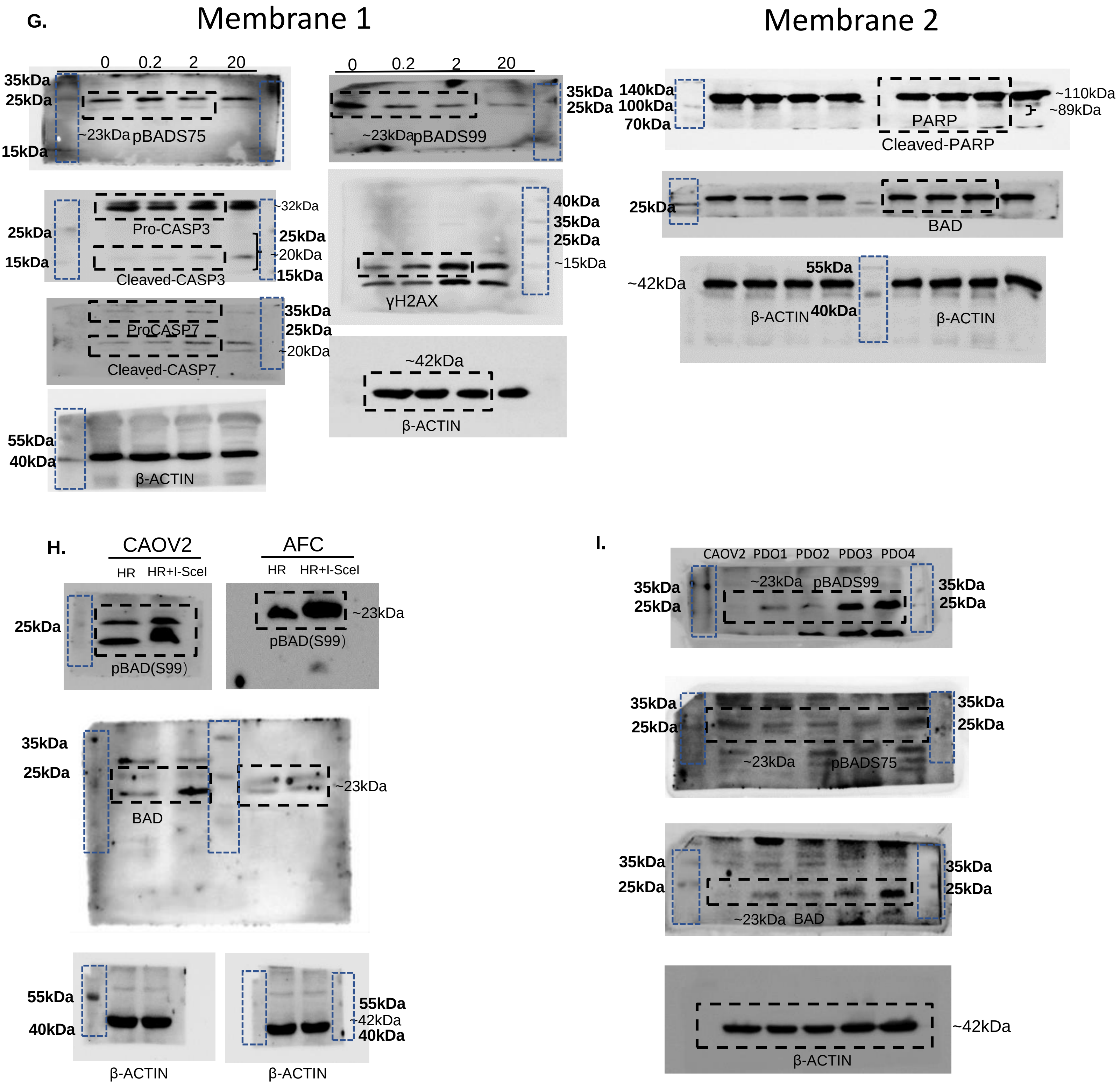

Supplementary Figure 11. Uncropped western blot images.

J.

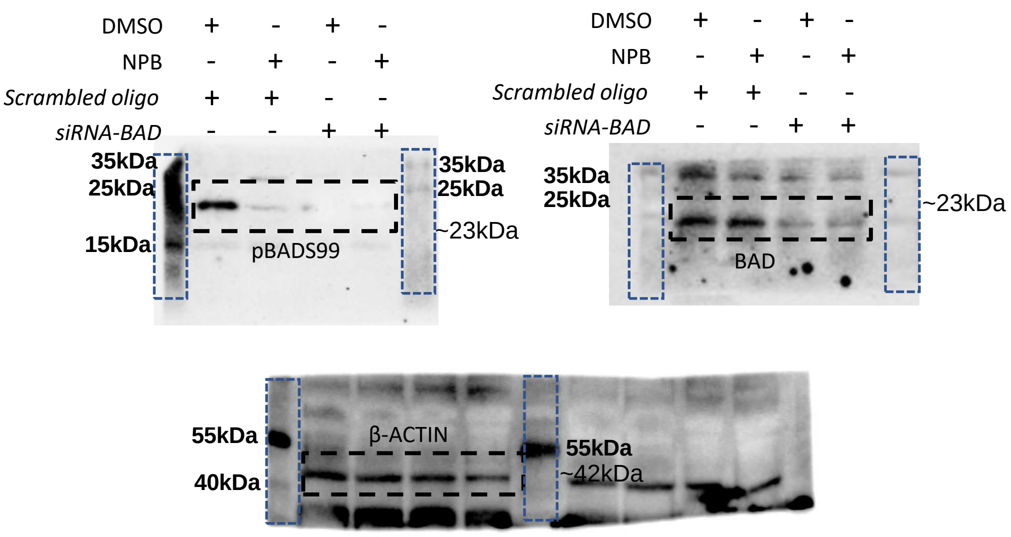

K.

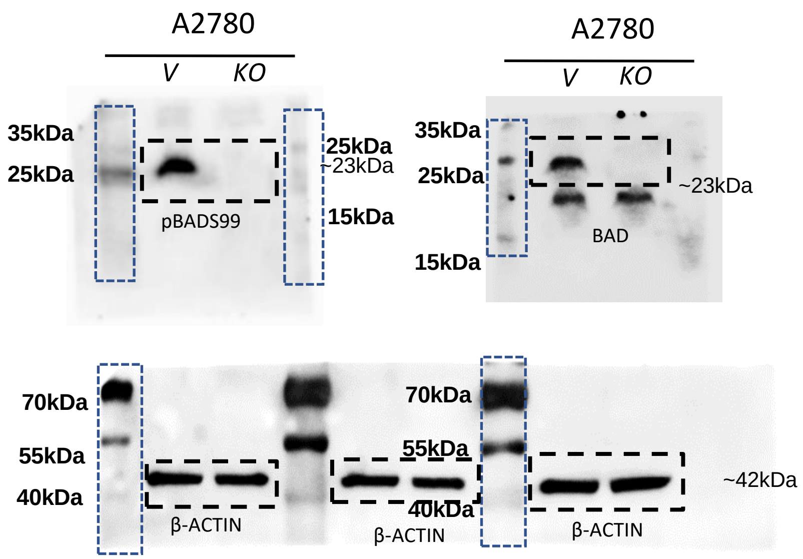

Supplementary Figure 11.

(A) Uncropped western blot images for figure 1C. All the proteins were probed on the two separate membranes which was sliced into multiple pieces and stripped with Western Blot Stripping Buffer. The blot was re-blocked and reprobed other antibodies and detected. Corresponding loading controls have been provided.

(B) Uncropped western blot images for figure 3E. All the proteins were probed on the two separate membranes which was sliced into multiple pieces and stripped with Western Blot Stripping Buffer. The blot was re-blocked and reprobed other antibodies and detected. Corresponding loading controls have been provided.

(C) Uncropped western blot images for figure 4C. All the proteins were probed on the two separate membranes which was sliced into multiple pieces and stripped with Western Blot Stripping Buffer. The blot was re-blocked and reprobed other antibodies and detected. Corresponding loading controls have been provided.

(D) Uncropped western blot images for Supplementary Figure 1G. All the proteins were probed on the two separate membranes which was sliced into multiple pieces. Corresponding loading controls have been provided.

(E) Uncropped western blot images for Supplementary Figure 3A. All the proteins were probed on the two separate membranes which was sliced into multiple pieces. Corresponding loading controls have been provided.

(F) Uncropped western blot images for Supplementary Figure 5D. Left: All the proteins were probed on the two separate membranes which was sliced into multiple pieces. Right: All the proteins were probed on the one membranes which was sliced into multiple pieces and stripped with Western Blot Stripping Buffer. The blot was re-blocked and reprobed other antibodies and detected. Corresponding loading controls have been provided. The heavy chain is denoted as HC. Immunoblotting is denoted as IB.

(G) Uncropped western blot images for Supplementary Figure 5E. All the proteins were probed on the two separate membranes which was sliced into multiple pieces and stripped

with Western Blot Stripping Buffer. The blot was re-blocked and reprobed other antibodies and detected. Corresponding loading controls have been provided.

(H) Uncropped western blot images for Supplementary Figure 5G. All the proteins were probed on the one membranes which was sliced into multiple pieces. Corresponding loading controls have been provided.

(I) Uncropped western blot images for Supplementary Figure 6C. All the proteins were probed on the one membranes which was sliced into multiple pieces and stripped with Western Blot Stripping Buffer. The blot was re-blocked and reprobed other antibodies and detected. Corresponding loading controls have been provided.

(J) Uncropped western blot images for Supplementary Figure 6G. All the proteins were probed on the one membranes which was sliced into multiple pieces. Corresponding loading controls have been provided.

(K) Uncropped western blot images for Supplementary Figure 8C. All the proteins were probed on the one membranes which was sliced into multiple pieces. Corresponding loading controls have been provided.

Supplementary Table 1.

Combination Index (CI) of NPB and PARP inhibitors (PARPis, Olaparib=OLA, Rucaparib=RUC, Talazoparib=TAL) is calculated in 8 cell models in constant ratio concentration (10-fold serial dilution)

	Doses																				
**Cell lines**	NPB (μM) / OLA (μM)	10^{-3}	10^{-2}	10^{-1}	10^{0}	10^{1}	10^{2}	NPB (μM) / RUC (μM)	10^{-3}	10^{-2}	10^{-1}	10^{0}	10^{1}	10^{2}	NPB (μM) / TAL (μM)	10^{-3}	10^{-2}	10^{-1}	10^{0}	10^{1}	10^{2}
**Caov2**		0.15	0.33	0.06	0.06	0.07	0.12		0.08	0.11	0.03	0.1	0.14	0.09		0.04	0.09	0.03	0.01	0.01	0.09
**OVCAR-3**		0.12	2.68	4.58	5.15	0.41	0.01		0.12	0.17	0.25	0.10	0.04	0.07		0.06	0.37	0.24	0.25	0.07	0.07
**OVCAR-433**		0.10	0.07	0.03	0.03	0.23	0.20		0.07	0.32	0.24	0.18	0.27	0.41		0.03	0.08	0.08	0.05	0.08	0.08
**HEYC2**		0.11	0.04	0.06	0.02	0.09	0.12		0.21	0.44	0.50	0.15	0.17	0.09		0.19	0.35	0.07	0.04	0.01	0.01
**Anglne**		0.14	0.11	0.13	0.06	0.40	0.14		0.03	0.02	0.02	0.01	0.04	0.40		0.02	0.03	0.04	0.03	0.04	0.04
**A2780**		1.29	0.10	0.25	0.08	0.05	0.48		0.14	0.10	0.08	0.16	0.07	0.42		0.03	0.25	0.04	0.06	0.02	0.21
**A2780CisR**		1.18	0.30	0.20	0.13	0.07	0.01		0.55	0.01	0.01	0.01	0.01	0.00		0.05	0.03	0.01	0.04	0.02	0.02
**AFC**		0.04	0.20	0.10	0.08	0.03	0.35		0.05	0.12	0.04	0.01	0.01	0.06		0.05	0.07	0.01	0.01	0.01	0.02

Supplementary Table 2.

IC_{50} was calculated in 8 cell lines treated with the indicated concentration of NPB and PARP inhibitor (PARPis, Olaparib=OLA, Rucaparib=RUC, Talazoparib=TAL) for 6 days. The presence of 1μM NPB led to a significant 10~400-fold decrease in PARP inhibitor IC_{50}.

Cell lines	IC_{50} ±SD (μM)						
	**NPB**	**OLA**	**OLA+NPB(1μM)**	**RUC**	**RUC+NPB(1μM)**	**TAL**	**TAL+NPB(1μM)**
**CAOV2**	2.0±0.35	1.34±0.21	0.013±0.01	1.14±0.33	0.018±0.002	0.24±0.05	0.005±0.0007
**OVCAR3**	0.66±0.2	2.19±0.80	0.022±0.02	1.48±0.36	0.054±0.012	2.16±0.48	0.24±0.056
**OVCAR433**	2.26±0.3	5.9±1.23	0.015±0.03	6.03±2.04	0.027±0.003	0.90±0.37	0.003±0.001
**HEYC2**	2.26±0.3	4.8±0.82	0.027±0.03	1.2±0.39	0.074±0.016	0.79±0.22	0.006±0.0008
**Anglne**	2.04±0.6	4.27±0.61	0.036±0.00	0.25±0.08	0.012±0.003	0.14±0.003	0.007±0.001
**A2780**	0.75±0.2	2.7±0.03	0.0264±0.00	1.63±0.42	0.025±0.005	0.96±0.003	0.007±0.001
**A2789CisR**	9.58±1.3	8.2±1.12	0.11±0.003	0.87±0.28	0.042±0.001	1.77±0.53	0.028±0.007
**AFC**	1.3±0.2	6.1±1.17	0.015±0.00	0.22±0.47	0.021±0.004	0.41±0.12	0.021±0.05

Supplementary Table 3.

Clinicopathologic and molecular profiling information of the cell lines used in this study.

Cells		Clinical pathology			Molecular profiling				
		Site	Age at diagnosis (years)	Ethnicity	PI3KCA	PTEN	P53	BRCA	KRAS
Epithelial Ovarian Cancers (EOC) cell lines	CAOV2[#] (OVCAR2)	Metastatic ascites	N.D	Caucasian	N.D.	N.D.	√.	N.D.	-
	OVCAR-3[#]	Metastatic ascites	60	Caucasian	-	-	√	-	-
	OVCAR-433*[#]	Metastatic ascites	N.D	Caucasian	-	-	√	BRCA2	-
	HEYC2* (HEYA8)	Human ovarian cancer xenograft	N.D	Caucasian	N.D.	N.D.	√	BRCA1	√
	Anglne[$]	primary	N.D	Caucasian	N.D.	N.D.	N.D.	N.D.	N.D.
	A2780[$]	primary	N.D	African	√	√	-	-	-
	A2780CisR*[$]	primary	N.D	African	√	√	-	BRCA1	-
Patient derived cancer cells	PDO-1 (AFC*[#])	Metastatic ascites	54	Asian	√	-	-	BRCA2	√
	PDO-2[$]	Primary	43	Asian	N.D.	N.D.	N.D.	N.D.	N.D.
	PDO-3[$]	primary	65	Asian	N.D.	N.D.	N.D.	N.D.	N.D.
	PDO-4[$]	primary	56	Asian	N.D.	N.D.	N.D.	N.D.	N.D.

N.D. not determined; * platinum resistant; [#] Metastatic ascites; [$] primary cell (PC); ascitic fluid cancer cells (AFC); Patient derived organoid (PDO). Cell lines gene profiling Figure was collected from website: https://cancer.sanger.ac.uk/cell_lines

Supplementary Table 4.

Cancer cell line source and 2D culture conditions.

Cells used in this study		Original histological subtype	Source	Culture medium
Epithelial Ovarian Cancers (EOC) cell lines	CAOV2[#] (OVCAR2)	HGS EOC	Kyoto U.	RPMI 1640, 10% FBS, 10 μg/ml insulin
	OVCAR-3[#]	HGS EOC	ATCC	RPMI 1640, 20% FBS
	OVCAR-433*[#]	Papillary serous cystadenocarcinoma	Kyoto U.	DMEM, 10% FBS
	HEYC2 (HEYA8)	HGS EOC	Kyoto U.	RPMI 1640, 10% FBS
	Anglne[$]	HGS EOC	DSMZ	RPMI 1640, 10% FBS
	A2780[$]	Endometrioid EOC	ECACC	RPMI 1640, 10% FBS
	A2780CisR*[$]	Endometrioid EOC	ECACC	RPMI 1640, 10% FBS,1μM cisplatin
Patient derived cancer cells	PDO-1 (AFC*[#])	HGS EOC	HKUSZH	Advanced DMEM(F12), 10% FBS, growth factors
	PDO-2[$]	HGS EOC	HKUSZH	Advanced DMEM(F12), 10% FBS, growth factors
	PDO-3[#]	HGS EOC	HKUSZH	Advanced DMEM(F12), 10% FBS, growth factors
	PDO-4[$]	HGS EOC	HKUSZH	Advanced DMEM(F12), 10% FBS, growth factors

Platinum-resistant; [#] ascitic fluid cancer cells (AFC); [$] primary cell (PC); epithelial ovarian cancer (EOC); high-grade serous (HGS); Patient derived organoid (PDO).
Kyoto U.:Dr. Noriomi Matsumura Kyoto University
Whereas cell lines were maintained with 10% FBS in the respective media, all experiments were performed with 2% FBS in the respective media.

Supplementary Table 5.

PARP inhibitor monotherapy efficacy and associated toxicities in clinical trials.

			**Objective response rate (ORR)**		**Toxicities**
**Study**	**PARPi**	**Ovarian Cancer**	**BRCA1/2 mutant**	**BRCA Wild type**	
Gelmon et al.	Olaparib	Plt-sensitive	60%	50%	Nausea (58-76%)<br>Fatigue (29-66%)<br>Vomiting (30-37%)<br>Diarrhea (21-33%)<br>Dysgeusia (27%)<br>Headache (20-25%)<br>Anemia (16-19%)<br>Neutropenia (5-9%)
		Plt-resistant	33%	4%	
Matulonis et al.	Olaparib	Plt-sensitive	48%	–	
		Plt-resistant	28%	–	
Oza et al.<br>ARIEL2/Study 10	Rucaparib	Plt-sensitive	66%	–	Nausea (75%)<br>Fatigue (69%)<br>Vomiting (37%)<br>Diarrhea (32%)<br>Dysgeusia (39%)<br>Anemia (19%)<br>Neutropenia (7%)
		Plt-resistant	25%	–	
Swisher et al.<br>ARIEL2	Rucaparib	Plt-sensitive	80%	29% (LOH high),<br>10% (LOH low)	
Bono et al.	Talazoparib	Plt-sensitive	55%	-	Nausea (49%)<br>Fatigue (50%)<br>Vomiting (25%)<br>Diarrhea (22%)<br>Alopecia (25%)<br>Headache (33%)<br>Anemia (16-19%)<br>Neutropenia (5-9%)<br>Thrombocytopenia (15%)
		Plt-resistant	20%	-	

PARPi: PARP inhibitor. Plt: platinum; LOH:LOH: loss of heterozygosity

Supplementary Table 6.

PARP inhibitor trapping capacities.

		Olaparib	Rucaparib	Talazoparib
Relative PARP-trapping capacity	PARP		++++	
	PARP1	+++		++++
	PARP2	++++		
Approvals		Ovarian/Breast (FDA/EMA/NMPA)	Ovarian (FDA/EMA)	Breast (FDA)

FDA: Food and Drug Administration; EMA: European medicine agency; NMPA (CFDA): National Medical Products Administration (China Food and Drug Administration)

Supplementary Table 7.

Combination therapy efficacy and associated toxicities between Olaparib and PI3K/AKT/mTOR or RAS/MEK/ERK signaling pathway inhibitors in ongoing clinical trials

Study (Trail)	Phase	Combination strategy	Population	Objective response rate (ORR)	Toxicities
NCT02208375	Ib	Olaparib+vistusertib (AZD2014)	Recurrent ovarian cancer (N=64)	20%	Nausea (84%)<br>Anemia (83%)<br>Hyperglycemia (76%)<br>Fatigue (73%)<br>increased creatinine (50%)<br>Vomiting (41%)<br>Diarrhea (39%)<br>Dysgeusia (27%)<br>Headache (42%)<br>leukopenia (55%)<br>thrombocytopenia (38%)
		Olaparib+capivasertib (AZD 5363)	Locally advanced recurrent endometrial adenocarcinoma<br>Recurrent HGSOC<br>gBRCAmut OC of any histology (N=30)	24%	Nausea (76%)<br>Anemia (89%)<br>Hyperglycemia (42%)<br>Fatigue (42%)<br>increased creatinine (58%)<br>Vomiting (39%)<br>Diarrhea (74%)<br>leukopenia (61%)<br>thrombocytopenia (18%)
NCT02338622 (COMPAKT)	I	Olaparib+capivasertib (AZD 5363)	Advanced ovarian cancer (N=25)	44%	
NCT01623349	I	Olaparib+buparlisib (BKM120)	Ovarian cancer (N=46)	29%	Nausea (78%)<br>Anemia (23%)<br>Hyperglycemia (39%)<br>Fatigue (65%)<br>increased creatinine (10%)<br>Dysgeusia (17%)<br>increased ALT (20%)<br>Vomiting (22%)<br>Diarrhea (35%)<br>leukopenia (12%)<br>thrombocytopenia (10%)
		Olaparib+alpelisib (BYL719)	Advanced ovarian cancer (N=34)	35.6%	Nausea (9.4%)<br>Hyperglycemia (15.6%)<br>increased ALT (9.4%)
NCT03162627	I	Olaparib+Selumetinib	Ovarian and Other Solid Tumors With Ras Pathway Alterations (N=12)	17%	Nausea (78%)<br>Anemia (79%)<br>Fatigue (64%)<br>increased creatinine (29%)<br>Dysgeusia (36%)<br>Diarrhea (50%)<br>leukopenia (21%)thrombocytopenia (7%)

Supplementary Table 8.

The CDSs of Flag-*h*BAD-WT, and -S99A.

cDNA	coding sequence (CDS)
hBAD-WT	5’**ATGGATTACAAGGACGACGATGACAAGGGCAGC**ATGTT<br>CCAGATCCCAGAGTTTGAGCCGAGTGAGCAGGAAGACTCCAGCTCTGCAG<br>AGAGGGGCCTGGGCCCCAGCCCCGCAGGGGACGGGCCCTCAGGCTCCGGC<br>AAGCATCATCGCCAGGCCCCAGGCCTCCTGTGGGACGCCAGTCACCAGCA<br>GGAGCAGCCAACCAGCAGCAGCCATCATGGAGGCGCTGGGGCTGTGGAGA<br>TCCGGAGTCGCCACAGCTCCTACCCCGCGGGGACGGAGGACGACGAAGGG<br>ATGGGGGAGGAGCCCAGCCCCTTTCGGGGCCGCTCGCGCTCGGCGCCCCCC<br>AACCTCTGGGCAGCACAGCGCTATGGCCGCGAGCTCCGGAGGATGAGTGA<br>CGAGTTTGTGGACTCCTTTAAGAAGGGACTTCCTCGCCCGAAGAGCGCGGG<br>CACAGCAACGCAGATGCGGCAAAGCTCCAGCTGGACGCGAGTCTTCCAGT<br>CCTGGTGGGATCGGAACTTGGGCAGGGGAAGCTCCGCCCCCTCCCAGTGA-<br>3’
hBAD-S99A	5’**ATGGATTACAAGGACGACGATGACAAGGGCAGC**ATGTT<br>CCAGATCCCAGAGTTTGAGCCGAGTGAGCAGGAAGACTCCAGCTCTGCAG<br>AGAGGGGCCTGGGCCCCAGCCCCGCAGGGGACGGGCCCTCAGGCTCCGGC<br>AAGCATCATCGCCAGGCCCCAGGCCTCCTGTGGGACGCCAGTCACCAGCA<br>GGAGCAGCCAACCAGCAGCAGCCATCATGGAGGCGCTGGGGCTGTGGAGA<br>TCCGGAGTCGCCACAGCTCCTACCCCGCGGGGACGGAGGACGACGAAGGG<br>ATGGGGGAGGAGCCCAGCCCCTTTCGGGGCCGCTCGCGC**GCC**GCGCCCC<br>CCAACCTCTGGGCAGCACAGCGCTATGGCCGCGAGCTCCGGAGGATGAGT<br>GACGAGTTTGTGGACTCCTTTAAGAAGGGACTTCCTCGCCCGAAGAGCGCG<br>GGCACAGCAACGCAGATGCGGCAAAGCTCCAGCTGGACGCGAGTCTTCCA<br>GTCCTGGTGGGATCGGAACTTGGGCAGGGGAAGCTCCGCCCCCTCCCAGTG<br>A-3’

Supplementary Table 9.

List of primary and secondary antibodies used.

Protein marker	Working dilution	Company/catalog number
**Phospho-BAD (Ser136)**	1:1000 (WB),<br>1:200 (IF)	Cell Signaling D25H8
**Phospho-BAD (Ser112)**	1:1000	Cell Signaling 40A9
**BAD (11E3)**	1:1000 (WB),<br>1:200 (IF),<br>1:100 (IP)	Cell Signaling 9268
**Phospho-BAD (Ser136) (IHC)**	1:200	GeneTex GTX79125
**BAD (IHC)**	1:200	GeneTex GTX50417
**BCL-2**	1:1000	Abcam ab32124
**BCL-W**	1:1000	Thermofisher PA5-86469
**BCL-XL**	1:1000	Abcam ab178844
**β-ACTIN**	1:1000	Santa Cruz sc-47778
**KI67**	1:1000 (WB),<br>1:200 (IHC)	Abcam ab16667
**CHK1**	1:1000	Cell Signaling 2G1D5
**phopho-CHK1 (Ser345)**	1:1000	Cell Signaling 133D3
**CCND1**	1:1000	Santa Cruz sc-8396
**CHK2**	1:1000	Cell Signaling D9C6
**phopho-CHK2(Thr383)**	1:1000	Abcam ab59408
**Caspase-7**	1:1000 (WB),<br>1:200 (IHC)	Santa Cruz B4-G2
**phospho-Histone H2A.X (Ser139)**	1:1000 (WB),<br>1:200 (IF)	Cell Signaling 2577S
**Caspase-3**	1:1000 (WB),<br>1:200 (IHC)	Santa Cruz sc-72721
**RAD51**	1:200	Abcam ab133534
**Anti-DDDDK Tag**	1:1000 (WB),<br>1:200 (IF)	Abbkine 1B10
**Anti-rabbit IgG (H+L), Biotinylated Antibody**	1:5000(WB)<br>1;1000(IHC)	Cell Signaling 14708
**Anti-mouse IgG (H+L), Biotinylated Antibody**	1:5000(WB)<br>1;1000(IHC)	Cell Signaling 14709
**IgG H&L (Alexa Fluor® 488)**	1:1000	Abcam ab150077
**IgG H&L (Alexa Fluor® 555)**	1:1000	Cell Signaling 4417

Supplementary Methods

The construction of *Flag-h*BAD-WT, and -S99A and transient transfection: Nucleotide mutations from TCG into GCC were introduced to the coding sequence (CDS) to achieve mutant *BAD-S99A* in which Ser99 was replaced with Ala. Flag-tag encoding DNA sequence (5-ATGGATTACAAGGACGACGATGACAAGGGCAGC-3) was added at the front 5' of the *hBAD-WT* and *hBAD-S99A CDS* to monitor the expression of exogenous BAD protein in cells. These *flag-hBAD* related cDNAs were then sub-cloned into pcDNA3.1(+) plasmid vector (with Nhe I (R0131, New England Biolabs (Beijing) Ltd., PRC) and EcoR I（R0101, New England Biolabs (Beijing) Ltd., PRC） restriction endonuclease. The CDSs of *flag-hBAD-WT and -S99A* were sequenced by Sangon Biotech (Shanghai, PRC) and are listed in SI 11. Constructs were designated as pcDNA3.1-flag-*h*BAD-WT and pcDNA3.1-flag-*h*BAD-S99A. Cells were transiently transfected using FuGENE® 6 Transfection Reagent (E2692, Promega, Madison, WI, USA) as per the manufacturer's instructions and as previously described (16).

HR reporter assay and sensitivity assays: To establish CAOV2-*DR-GFP* and AFC-*DR-GFP* cells, the *pDR-GFP* plasmid was stably integrated into CAOV2 and AFC cell lines as previously described (17). After 48 hours of pretreatment with NPB, CAOV2-*DR-GFP* and AFC-*DR-GFP* (1×10^6) cells were transfected by electroporation using FuGENE HD (Promega, US) with 2μg pCMV-I-SceI, respectively. The cells were assessed for green fluorescence emission using flow cytometry. Results were corrected according to the transfection efficiency of the cell line.

Apoptotic DNA Laddering: Cells were collected by centrifugation, and the cell pellets were lysed by gentle, repeated pipetting in a 2-mL microcentrifuge tube with 0.5 mL cell lysis buffer (50 mM Tris-HCl, pH 7.5, 0.5% Triton X-100, 20 mM EDTA). After centrifugation at 12000 rpm for 10 min to remove cell debris, as well as intact nuclei of non-apoptotic cells, the supernatant was subject to one round of phenol:chloroform:isoamyl alcohol (25:24:1; pH 7.4; 0.5mL) extraction. Apoptotic DNA fragments and contaminating RNA present in the liquid phase were precipitated by adding 50μL 3M sodium acetate and 50μL phenol:chloroform:isoamyl alcohol (25:24:1; pH 7.4; 0.5 mL). After incubation on ice for 5 min, the precipitated nucleic acids were pelleted by centrifugation at 12000rpm for 10 min. The pellet was resuspended in RNaseA buffer (Promega, Madison, WI, USA) by incubation at 37°C for 30 min or longer. After centrifugation at 12000rpm for 10 min, the pellet was resuspended in phenol:chloroform: isoamyl alcohol (25:24:1; pH 7.4; 0.5mL), and this step was repeated. Apoptotic DNA fragments were resolved by 2 % TAE agarose gel electrophoresis (17, 18). Genomic DNA was extracted following Wizard® Genomic DNA Purification Kit instructions (Promega, Madison, WI, USA).

***siRNA* transfection and *BAD*-knockout stable cell line establishment**: The target-directed *siRNAs*

(*shRNA-BAD1*, 5′-GCUCCGCACCAUGAGUGACGAGUUU-3′ and *shRNA-BAD2*, 5′-AAACUCGUCACUCAUCCUCCGGAGC-3′) and the negative control *siRNA*, which was used for normalization, were synthesized by GENEWIZ (Suzhou, China). After seeding and adherence for 24h, cells were washed with PBS and transfected with the *siRNAs* for 24h using FuGENE HD (Promega, US) according to the manufacturer's instructions. The siRNAs were transfected either separately with constant-ratio diluted PARP inhibitor (single treatments) or with combinations of constant-ratio diluted PARP inhibitor and NPB(1μM). The developed CRISPR/Cas9 system was employed to delete BAD in A2780 cells. sgRNA Sequences targeting BAD (GENEWIZ, Suzhou, China) cloned into the pSpCas9(BB)-2A-Puro (PX459) V2.0 construct (#62988, Addgene, Cambridge, MA, USA) were listed as followed:

#1 sgRNA 5′- *CACCGCCACAGCTCCTACCCCGCGGTTT*-3′

#2 sgRNA 5′- *CACCGGTGACTGGCGTCCCACAGGGTTT*-3′

#3 sgRNA 5′- *CACCGAGCGCTATGGCCGCGAGCTCGTTT*-3′

#4 sgRNA 5′- *CACCGCTCCGGCAAGCATCATCGCCGTTT*-3′

#5 sgRNA 5′- CACCGTTCGTCGTCCTCCGTCCCCGGTTT -3′

BAD co-immunoprecipitation (co-IP): BAD co-immunoprecipitation (co-IP) assay was performed using BAD-antibody conjugated magnetic beads from Universal IP/Co-IP Toolkit (Abbikine) as per manufacturer's instructions (Cat #: KTD104-EN, Abbikine, China).

Supplementary References

1. Pandey V, *et al.* (2018) Discovery of a small-molecule inhibitor of specific serine residue BAD phosphorylation. *Proc. Natl. Acad. Sci. U. S. A.* 115(44):E10505.
2. Tapodi A, *et al.* (2005) Pivotal role of Akt activation in mitochondrial protection and cell survival by poly(ADP-ribose) polymerase-1 inhibition in oxidative stress. *Journal of Biological Chemistry* 280(42):35767-35775.
3. Veres B, *et al.* (2003) Decrease of the inflammatory response and induction of the Akt/protein kinase B pathway by poly-(ADP-ribose) polymerase 1 inhibitor in endotoxin-induced septic shock. *Biochemical pharmacology* 65(8):1373-1382.
4. Veres B, *et al.* (2004) Regulation of kinase cascades and transcription factors by a poly(ADP-ribose) polymerase-1 inhibitor, 4-hydroxyquinazoline, in lipopolysaccharide-induced inflammation in mice. *Journal of Pharmacology and Experimental Therapeutics* 310(1):247-255.
5. Sun C, *et al.* (2017) Rational combination therapy with PARP and MEK inhibitors capitalizes on therapeutic liabilities in RAS mutant cancers. *Science translational medicine* 9(392).
6. Tan Y, Ruan H, Demeter MR, & Comb MJ (1999) p90(RSK) blocks bad-mediated cell death via a protein kinase C-dependent pathway. *J Biol Chem* 274(49):34859-34867.
7. Manning BD & Cantley LC (2007) AKT/PKB signaling: navigating downstream. *Cell* 129(7):1261-1274.
8. Harada H, Andersen JS, Mann M, Terada N, & Korsmeyer SJ (2001) p70S6 kinase signals cell survival as well as growth, inactivating the pro-apoptotic molecule BAD. *Proc Natl Acad Sci U S A* 98(17):9666-9670.
9. Jiang P, Du W, & Wu M (2007) p53 and Bad: remote strangers become close friends. *Cell Res* 17(4):283-285.
10. Adachi M & Imai K (2002) The proapoptotic BH3-only protein BAD transduces cell death signals independently of its interaction with Bcl-2. *Cell Death Differ* 9(11):1240-1247.
11. Remmele W & Stegner HE (1987) [Recommendation for uniform definition of an immunoreactive score (IRS) for immunohistochemical estrogen receptor detection (ER-ICA) in breast cancer tissue]. *Pathologe* 8(3):138-140.
12. Maenhoudt N, *et al.* (2020) Developing Organoids from Ovarian Cancer as Experimental and Preclinical Models. *Stem Cell Reports* 14(4):717-729.
13. Kopper O, *et al.* (2019) An organoid platform for ovarian cancer captures intra- and interpatient heterogeneity. *Nat Med* 25(5):838-849.
14. Boehnke K, *et al.* (2016) Assay Establishment and Validation of a High-Throughput Screening Platform for Three-Dimensional Patient-Derived Colon Cancer Organoid Cultures. *J Biomol Screen* 21(9):931-941.
15. Zhao H, *et al.* (2019) Sphereforming assay vs. organoid culture: Determining longterm stemness and the chemoresistant capacity of primary colorectal cancer cells. *Int J Oncol* 54(3):893-904.
16. Pandey V, *et al.* (2018) Discovery of a small-molecule inhibitor of specific serine residue BAD phosphorylation. *Proceedings of the National Academy of Sciences of the United States of America* 115(44):E10505-E10514.
17. Pierce AJ, Johnson RD, Thompson LH, & Jasin M (1999) XRCC3 promotes homology-directed repair of DNA damage in mammalian cells. *Genes Dev* 13(20):2633-2638.
