# Supplementary material for: Combined inhibition of BADSer99 phosphorylation and PARP ablates models of recurrent ovarian carcinoma
Source: Commun Med (Lond). 2022 Jul 2;2:82. doi: 10.1038/s43856-022-00142-3 (PMC9250505; doi:10.1038/s43856-022-00142-3)
Supplement: Supplementary file 2 — Description of Additional Supplementary Files [file 43856_2022_142_MOESM2_ESM.pdf]

Description of Additional Supplementary Files

File Name: Supplementary Data 1

Description: List of clinical tissue samples.

File Name: Supplementary Data 2

Description: All source data underlying the graphs and charts
