# Supplementary material for: Combined inhibition of BADSer99 phosphorylation and PARP ablates models of recurrent ovarian carcinoma
Source: Commun Med (Lond). 2022 Jul 2;2:82. doi: 10.1038/s43856-022-00142-3 (PMC9250505; doi:10.1038/s43856-022-00142-3)
Supplement: Supplementary file 6 — Reporting Summary [file 43856_2022_142_MOESM6_ESM.pdf]

nature research

Corresponding author(s): Peter E. Lobie, and Vijay Pandey

Last updated by author(s): Jan 7, 2022

Reporting Summary

Nature Research wishes to improve the reproducibility of the work that we publish. This form provides structure for consistency and transparency in reporting. For further information on Nature Research policies, see our Editorial Policies and the Editorial Policy Checklist.

Statistics

For all statistical analyses, confirm that the following items are present in the figure legend, table legend, main text, or Methods section.

n/a	Confirmed	
☐	☒	The exact sample size (n) for each experimental group/condition, given as a discrete number and unit of measurement
☐	☒	A statement on whether measurements were taken from distinct samples or whether the same sample was measured repeatedly
☐	☒	The statistical test(s) used AND whether they are one- or two-sided<br>*Only common tests should be described solely by name; describe more complex techniques in the Methods section.*
☒	☐	A description of all covariates tested
☐	☒	A description of any assumptions or corrections, such as tests of normality and adjustment for multiple comparisons
☐	☒	A full description of the statistical parameters including central tendency (e.g. means) or other basic estimates (e.g. regression coefficient) AND variation (e.g. standard deviation) or associated estimates of uncertainty (e.g. confidence intervals)
☐	☒	For null hypothesis testing, the test statistic (e.g. F, t, r) with confidence intervals, effect sizes, degrees of freedom and P value noted<br>*Give P values as exact values whenever suitable.*
☒	☐	For Bayesian analysis, information on the choice of priors and Markov chain Monte Carlo settings
☒	☐	For hierarchical and complex designs, identification of the appropriate level for tests and full reporting of outcomes
☐	☒	Estimates of effect sizes (e.g. Cohen's d, Pearson's r), indicating how they were calculated

Our web collection on statistics for biologists contains articles on many of the points above.

Software and code

Policy information about availability of computer code

Data collection	Nikon Elements (NIS ElementsAR ver. 4.6.0.) and Zeiss Zen software (2012 S4) were used to acquire images
Data analysis	ImageJ2/FIJI was used to contrast and overlay images as described in the Methods section.<br>SPSS 25 (IBMSPSS Statistics, IBM Corp., Armonk, NY, USA) was used to analyze data from clinical patients. The codes that support the fundings of this study are available from corresponding author upon reasonable request.<br>GraphPad Prism 7.0 (GraphPad Software, San Diego, CA, USA) was used for Statistical analysis in vitro assays and in vivo assay. The codes that support the fundings of this study are available from corresponding author upon reasonable request.

For manuscripts utilizing custom algorithms or software that are central to the research but not yet described in published literature, software must be made available to editors and reviewers. We strongly encourage code deposition in a community repository (e.g. GitHub). See the Nature Research guidelines for submitting code & software for further information.

Data

Policy information about availability of data

All manuscripts must include a data availability statement. This statement should provide the following information, where applicable:

- Accession codes, unique identifiers, or web links for publicly available datasets
- A list of figures that have associated raw data
- A description of any restrictions on data availability

All raw and processed data will be made available upon request.

Field-specific reporting

Please select the one below that is the best fit for your research. If you are not sure, read the appropriate sections before making your selection.

☒ Life sciences ☐ Behavioural & social sciences ☐ Ecological, evolutionary & environmental sciences

For a reference copy of the document with all sections, see nature.com/documents/nr-reporting-summary-flat.pdf

Life sciences study design

All studies must disclose on these points even when the disclosure is negative.

Sample size	For all cell culture experiments requiring statistical analysis, n=3 was chosen as the minimal replicate number as indicated in the figure legends. For animal experiments requiring statistical analysis we used 6 animals per group.
Data exclusions	Data were not excluded from analysis
Replication	All experiments were reliably reproduced, detailed statistical analysis is provided.
Randomization	All cells and all sections that passed quality control used for analyzing were selected randomly and equally. No specific method of randomization was used for generation of samples or in animal experiments. The animals were littermate, and inbred lines were used, where the individual mice were identical. The animals were randomly assigned to the experimental conduct
Blinding	Analysis of data was performed by blinded researchers.

Reporting for specific materials, systems and methods

We require information from authors about some types of materials, experimental systems and methods used in many studies. Here, indicate whether each material, system or method listed is relevant to your study. If you are not sure if a list item applies to your research, read the appropriate section before selecting a response.

Materials & experimental systems

n/a	Involved in the study	
☐	☒	Antibodies
☐	☒	Eukaryotic cell lines
☒	☐	Palaeontology and archaeology
☐	☒	Animals and other organisms
☐	☒	Human research participants
☒	☐	Clinical data
☒	☐	Dual use research of concern

Methods

n/a	Involved in the study	
☒	☐	ChIP-seq
☐	☒	Flow cytometry
☒	☐	MRI-based neuroimaging

Antibodies

Antibodies used

As described in SI text (G):
Phospho-BAD (Ser136) Cell Signaling D25H8
Phospho-BAD (Ser112) Cell Signaling 40A9
BAD (11E3) Cell Signaling 9268
Phospho-BAD (Ser136) (IHC) GeneTex GTX79125
BAD (IHC) GeneTex GTX50417
β-ACTIN Santa Cruz sc-47778
KI67 Abcam ab16667
CHK1 Cell Signaling 2G1D5
phopho-CHK1 (Ser345) Cell Signaling 133D3
CCND1 Santa Cruz sc-8396
CHK2 Cell Signaling D9C6
phopho-CHK2(Thr383) Abcam ab59408
Caspase-7 Santa Cruz B4-G2
phospho-Histone H2A.X (Ser139) Cell Signaling 2577S
Cleaved CASP3 Santa Cruz sc-72721
RAD51 Abcam ab133534
Anti-DDDDK Tag Abbkine 1B10
IgG H&L (Alexa Fluor® 488) Abcam ab150077
IgG H&L (Alexa Fluor® 555) Cell Signaling 4417

Validation

This antibody was also validated in our previous work or other published work.

Validation	work (Shekhar and Lapan, 2016), where it was shown to specifically label rod bipolar cells based on overlap with rod bipolar cellspecific markers that were identified in Drop-seq data.

Eukaryotic cell lines

Policy information about cell lines

Cell line source(s)	We summarized all the cancer cell lines source.
Authentication	All the cell lines have been authenticated and have STR identification certificate.
Mycoplasma contamination	Yes, any mycoplasma contamination has been excluded.
Commonly misidentified lines (See ICLAC register)	No commonly misidentified cell lines were used

Animals and other organisms

Policy information about studies involving animals; ARRIVE guidelines recommended for reporting animal research

Laboratory animals	BALB/C nude mice
Wild animals	No wild animals were used in this study.
Field-collected samples	No field-collected samples were used in this study.
Ethics oversight	The mouse work was performed according to guidelines for the care and use of laboratory animals, approved by the Laboratory Animal Ethics Committee (Certificate number: YW) at Peking University, and ethical approval obtained from Tsinghua Shenzhen International Graduate School (Number:9, Year 2020).

Note that full information on the approval of the study protocol must also be provided in the manuscript.

Human research participants

Policy information about studies involving human research participants

Population characteristics	The human tissue samples used herein consisted of 80 EOC and 20 specimens of NCO from patients that underwent surgery at the University of Hong Kong-Shenzhen Hospital (HKU-SZH, Shenzhen, Guangdong, China) between 2016 and 2018. Clinical information and follow-up data were obtained from the hospital medical records. The NCO tissue was collected from ovaries of patients with uterine fibroids (non-ovarian pathology) who elected to undergo concomitant salpingo-oophorectomy. All cancer patients were staged according to the International Federation of Gynecology and Obstetrics (FIGO) classification.
Recruitment	The human tissue samples used herein consisted of 80 EOC and 20 specimens of NCO from patients that underwent surgery at the University of Hong Kong-Shenzhen Hospital (HKU-SZH, Shenzhen, Guangdong, China) between 2016 and 2018.
Ethics oversight	The use of patient specimens in this study was approved by the Institutional Ethical Committee of the HKU-SZH (certificate No. hkuszh2019105, approval No.[illegible] [2019] 096, Date: 2019.03.26). Patient consent forms were obtained from all patients in accordance with the declaration of Helsinki.

Note that full information on the approval of the study protocol must also be provided in the manuscript.

Flow Cytometry

Plots

Confirm that:

☒ The axis labels state the marker and fluorochrome used (e.g. CD4-FITC).

☒ The axis scales are clearly visible. Include numbers along axes only for bottom left plot of group (a 'group' is an analysis of identical markers).

☐ All plots are contour plots with outliers or pseudocolor plots.

☒ A numerical value for number of cells or percentage (with statistics) is provided.

Methodology

Sample preparation	Phosphatidylserine exposure and cell death were assessed using Annexin-V-FLUOS and PI-stained (Neobioscience, Shenzhen, China) cells as described previously. Prior to acquisition, cell pellets were washed with PBS and stained with Annexin V and PI at least 50,000-10,0000 cells per sample were recorded.
Instrument	CytoFLEX (Beckman Coulter, Inc. USA)

Software	CytExpert Acquisition and Analysis Software Version 2.4
Cell population abundance	Not applicable
Gating strategy	Initial gates - FSC-A/SSC-A to discriminate cells from debris; then cells were gated in FSC-W/FSC-A to discriminate single cells; then cells were gated in SSC-W/SSC-A to discriminate live cells. Resulted population were analyzed on SSC-A/FL1 to find cells stained with Alexa Fluor 488. Mock transected cells were used for selecting negative population.

☒ Tick this box to confirm that a figure exemplifying the gating strategy is provided in the Supplementary Information.
